# Non-Transcriptional and Translational Function of Canonical NF-*κ*B Signaling in Activating ERK1/2 in IL-1*β*-Induced COX-2 Expression in Synovial Fibroblasts

**DOI:** 10.3389/fimmu.2020.579266

**Published:** 2020-10-07

**Authors:** Rei Nakano, Taku Kitanaka, Shinichi Namba, Nanako Kitanaka, Yoko Suwabe, Tadayoshi Konno, Jun Yamazaki, Tomohiro Nakayama, Hiroshi Sugiya

**Affiliations:** ^1^ Laboratory for Cellular Function Conversion Technology, RIKEN Center for Integrative Medical Sciences, Yokohama, Japan; ^2^ Laboratory of Veterinary Radiology, Department of Veterinary Medicine, College of Bioresource Sciences, Nihon University, Fujisawa, Japan; ^3^ Laboratory of Veterinary Biochemistry, Department of Veterinary Medicine, College of Bioresource Sciences, Nihon University, Fujisawa, Japan; ^4^ Laboratory of Veterinary Pharmacology, Department of Veterinary Medicine, College of Bioresource Sciences, Nihon University, Fujisawa, Japan

**Keywords:** rheumatoid arthritis, interleukin 1*β* (IL-1β), cyclooxygenase 2 (COX-2), NF-*κ*B—nuclear factor kappa B, ERK1/2

## Abstract

The pro-inflammatory cytokine interleukin 1*β* (IL-1*β*) induces the synthesis of prostaglandin E_2_ by upregulating cyclooxygenase-2 (COX-2) in the synovial tissue of individuals with autoimmune diseases, such as rheumatoid arthritis (RA). IL-1*β*-mediated stimulation of NF-*κ*B and MAPK signaling is important for the pathogenesis of RA; however, crosstalk(s) between NF-*κ*B and MAPK signaling remains to be understood. In this study, we established a model for IL-1*β*-induced synovitis and investigated the role of NF-*κ*B and MAPK signaling in synovitis. We observed an increase in the mRNA and protein levels of COX-2 and prostaglandin E_2_ release in cells treated with IL-1*β*. NF-*κ*B and ERK1/2 inhibitors significantly reduced IL-1*β*-induced COX-2 expression. IL-1*β* induced the phosphorylation of canonical NF-*κ*B complex (p65 and p105) and degradation of I*κ*B*α*. IL-1*β* also induced ERK1/2 phosphorylation but did not affect the phosphorylation levels of p38 MAPK and JNK. IL-1*β* failed to induce COX-2 expression in cells transfected with siRNA for p65, p105, ERK1, or ERK2. Notably, NF-*κ*B inhibitors reduced IL-1*β*-induced ERK1/2 phosphorylation; however, the ERK1/2 inhibitor had no effect on the phosphorylation of the canonical NF-*κ*B complex. Although transcription and translation inhibitors had no effect on IL-1*β*-induced ERK1/2 phosphorylation, the silencing of canonical NF-*κ*B complex in siRNA-transfected fibroblasts prevented IL-1*β*-induced phosphorylation of ERK1/2. Taken together, our data indicate the importance of the non-transcriptional/translational activity of canonical NF-*κ*B in the activation of ERK1/2 signaling involved in the IL-1*β*-induced development of autoimmune diseases affecting the synovial tissue, such as RA.

## Introduction

Rheumatoid arthritis (RA) is a chronic and progressive inflammatory autoimmune disease characterized by a dysregulated immune system in the synovial joint membrane, thereby causing severe damage and destruction of the cartilage and bone. Synovitis (inflammation of the synovial membrane) is a hallmark of RA. Pro-inflammatory cytokines, such as interleukin 1*β* (IL-1*β*) and tumor necrosis factor α (TNF-α), play a crucial role in the development of synovitis and progressive joint destruction (1, 2). The use of antibodies against IL-1 to treat patients with RA improves symptoms associated with RA and reduces joint erosions (3, 4). IL-1*β* induces the expression of prostaglandin E_2_ by upregulating cyclooxygenase-2 (COX-2) in pro-inflammatory conditions. IL-1*β* activates numerous cellular signaling pathways, including nuclear factor-*κ*B (NF-*κ*B) and mitogen-activated protein kinase (MAPK) signaling.

NF-*κ*B is a transcription factor that is important in regulating immune response and inflammation (5, 6). NF-*κ*B consists of homo- and heterodimers of the Rel family of proteins, such as RelA (p65), RelB, c-Rel, p105/p50, and p100/p52 (6). The activity of NF-*κ*B is primarily regulated by its interaction with inhibitory proteins, such as I*κ*B, thereby enabling the cytoplasmic retention of these inactive complexes. NF-*κ*B is activated upon the degradation of I*κ*B that disrupts the interaction between I*κ*B and NF-*κ*B. Subsequently, the released NF-*κ*B translocates into the nucleus where it induces the expression of immune and inflammatory genes by binding to their promoters (6). In response to the pro-inflammatory cytokine IL-1*β*, the heterotrimeric complex of NF-*κ*B comprising p50, RelA (p65), and I*κ*Bα gets activated and results in canonical NF-*κ*B signaling (5, 6).

MAPK signaling pathways are involved in the regulation of various cellular functions, including inflammation. MAPKs are serine-threonine kinases, such as c-Jun NH_2_-terminal kinase (JNK), p38 MAPK, and extracellular signal-regulated kinase (ERK); all these kinases exist as isoforms in mammals. Depending on the stimulus and cell type, MAPKs are activated *via* multiple pathways, thereby phosphorylating a wide range of substrates, such as transcription factors and cytoskeletal proteins, and resulting in specific cellular responses (7–13). The MAPK signaling cascades consist of at least three sequential kinase components: MAPK kinase kinase, MAPK kinase, and MAPK. MAPK kinase kinases phosphorylate the serine/threonine residues of and activate MAPK kinases that phosphorylate the threonine/tyrosine residues in the activation loop of MAPKs, thereby stimulating MAPKs (14, 15). MAPK signaling activates NF-*κ*B in synovial fibroblasts in other cellular models (16–21). Pro-inflammatory cytokines (*e.g.* IL-1*β* and TNF-α) stimulate the activation of NF-*κ*B and MAPK signaling in synovial fibroblasts; however, the correlation between NF-*κ*B and MAPK signaling remains to be elucidated (8, 9).

In this study, we have investigated IL-1*β*-induced expression of COX-2 and its role in the synthesis of prostaglandin E_2_ in canine synovial fibroblasts. We observed crosstalk between NF-*κ*B and ERK1/2 MAPK signaling. Canonical NF-*κ*B signaling induced the activation of ERK1/2 in the presence of transcription and translation inhibitors. Taken together, our findings indicate that the non-transcriptional/translational function of canonical NF-*κ*B signaling regulates the activation of ERK1/2 signaling in synovial fibroblasts.

## Materials and Methods

### Materials

TRIzol and Lipofectamine 2000 were obtained from Life Technologies Co. (Carlsbad, CA). The Thermal Cycler Dice Real Time System II, TP900 DiceRealTime v4.02B, SYBR Premix Ex Taq II, PrimeScript RT Master Mix, and CELLBANKER 1 plus medium were purchased from TaKaRa Bio Inc. (Shiga, Japan). Rabbit monoclonal antibodies against human total JNK (t-JNK, EPR140 (2), Cat# ab110724, RRID : AB_10866293) and COX-1 (EPR5867, Cat# ab133319, RRID : AB_11157915), and rabbit polyclonal antibody against COX-2 (Cat# ab102005, RRID : AB_10972360) were procured from Abcam (Cambridge, UK). Anti-human phosphorylated p65 (Ser536) (p-p65, 93H1, Cat# 3033, RRID : AB_331284), anti-human total p65 (t-p65, D14E12, Cat# 8242, RRID : AB_10859369), anti-human total-I*κ*Bα (t-I*κ*Bα, I44D4, Cat# 4812, RRID : AB_10694416), anti-human phosphorylated p105 (Ser 933) (p-p105, 18E6, Cat# 4806, RRID : AB_2282911), anti-human total p105 (t-p105, D7H5M, Cat# 12540, RRID : AB_2687614), anti-rat total-ERK1/2 (t-ERK1/2, 137F5, Cat# 4695, RRID : AB_390779), anti-human phospho-ERK1/2 (Thr202/Tyr204) (p-ERK1/2, D13.14.4E, Cat# 4370, RRID : AB_2315112), anti-human total-p38 (t-p38, D13E1, Cat# 8690, RRID : AB_10999090), and anti-human phospho-p38 (Thr180/Tyr182) (p-p38, 3D7, Cat# 9215, RRID : AB_331762) rabbit monoclonal or polyclonal antibodies, horseradish peroxidase-conjugated anti-mouse (Cat# 7076, RRID : AB_330924) and anti-rabbit IgG antibodies (Cat# 7074, RRID : AB_2099233) were obtained from Cell Signaling Technology Japan, K.K. (Tokyo, Japan). Rabbit polyclonal antibody against phospho-JNK (Thr183/Tyr185) (p-JNK, Cat# V7931, RRID : AB_430864) was purchased from Promega, Co. (Madison, WI). BAY11-7082, TPCA-1, SKF86002, U0126, FR180204, SP600125, actinomycin D, cycloheximide, and mouse monoclonal anti-β-actin antibody (AC74, Cat# A5441, RRID : AB_476744) were procured from Sigma-Aldrich Inc. (St Louis, MO). ImageQuant LAS 4000 mini, protein A plus G Sepharose, and ECL Western blotting Analysis System were obtained from GE Healthcare (Piscataway, NJ). Mini-PROTEAN TGX gel and polyvinylidene difluoride membranes were purchased from Bio-Rad (Hercules, CA). Block Ace and Complete mini EDTA-free protease inhibitor mixture were purchased from Roche (Mannheim, Germany). Dulbecco’s modified Eagle’s medium supplemented with 1 g/L glucose (DMEM-LG) was obtained from Wako Pure Chemical Industries, Ltd. (Osaka, Japan). An enzyme-linked immunosorbent assay kit for prostaglandin E_2_ was procured from Cayman Chemical Co. (Ann Arbor, MI). Canine recombinant IL-1*β* was purchased from Kingfisher Biotech, Inc. (Saint Paul, MN). StatMate IV was obtained from ATMS (Tokyo, Japan). A freezing vessel (BICELL) was procured from Nihon Freezer Co., Ltd. (Tokyo, Japan).

### Cell Culture

Canine synovial fibroblasts isolated from the synovium of the stifle joint were a kind gift from Ms. Aki Ohmori, Teikyo University School of Medicine. We used flow cytometry to characterize cells by their surface markers: positive for fibroblast markers CD29 (97.86 ± 1.23%), CD44 (97.40 ± 1.30%), and CD90 (97.50 ± 1.42%), and negative for hematopoietic cell markers CD14 (1.60 ± 0.50%), CD34 (1.12 ± 0.10%), CD45 (0.97 ± 0.13%), and HLA-DR (2.73 ± 1.45%) (9). Dissociated cells were maintained in static culture in DMEM-LG supplemented with 10% fetal bovine serum (FBS) in a 5% CO_2_ incubator at 37°C. The medium was replaced once a week. Cells were cryopreserved and thawed as previously described (7–13, 22–26). Briefly, cells were harvested using 0.25% trypsin-EDTA once they were 90–95% confluent and resuspended in CELLBANKER 1 plus medium at a density of 2 × 10^6^ cells/500 μl. The cell suspension (500 μl) was placed into sterilized serum tubes that were placed in a freezing vessel (BICELL) and cryopreserved at −80°C. Before performing the experiment, tubes were removed from the BICELL vessel and immersed in a water bath at 37°C. The thawed cell suspension was transferred into a centrifuge tube containing DMEM-LG with 10% FBS and centrifuged at 300×*g* for 3 min. The pellet was resuspended in DMEM-LG containing 10% FBS and transferred to a 75 cm^2^ culture flask. Static cultures were maintained under the same conditions as prior to cryopreservation. Cells were harvested using 0.25% trypsin-EDTA once they were ~90% confluent; the collected cells were seeded at a density of 1 × 10^6^ cells per 75 cm^2^ culture flask. Experiments were performed with canine synovial fibroblasts from the fourth passage. Each experiment was performed with cells derived from a single donor.

### Quantitative Reverse Transcription-Polymerase Chain Reaction

RT-qPCR was performed as previously described (7–13, 22–26). Total RNA was extracted from canine synovial fibroblasts using TRIzol. First-strand cDNA synthesis was performed using 500 ng of total RNA with the PrimeScript RT Master Mix. Real-time PCR was performed using 2 µl of the first-strand cDNA, SYBR Premix Ex Taq II, and primers specific for COX-1, COX-2, and TBP (TATA-binding protein; housekeeping internal control) in a total reaction volume of 25 μl (**Table 1**). Real-time PCR for no-template control was performed using 2 µl of RNase- and DNA-free water. Additionally, real-time PCR for the control for reverse transcription was performed using 2 µl of the RNAs. PCR was performed using the Thermal Cycler Dice Real Time System II with the following protocol: one cycle of denaturation at 95°C for 30 s, 40 cycles of denaturation at 95°C for 5 s, and annealing/extension at 60°C for 30 s. Data were analyzed using the second derivative maximum and comparative cycle threshold (ΔΔCt) methods using the real-time PCR analysis software. TBP amplification from the same amount of cDNA was used as the endogenous control, while amplification from feline synovial fibroblasts at 0 h was used as the calibration standard.

**Table 1:** Primer sequences for RT-qPCR.

Gene Name	Gene bank ID	Primer sequences
*COX-2*	NM_001003354.1	F: 5′-TGTGTCTCATTAACCTGCATGTACC-3′
		R: 5′-CAGTGATATTTGCACCTGTGTCCTC-3′
*COX-1*	NM_001003023.2	F: 5′-ACGTGGCTGTGGAAACCATC-3′
		R: 5′-GGCATCAATGTCTCCATACAGCTC-3′
*TBP*	XM_863452	F: 5′-ACTGTTGGTGGGTCAGCACAAG-3′
		R: 5′-ATGGTGTGTACGGGAGCCAAG-3′

### Western Blotting

Western blotting was performed as previously described (7–13, 22–26). Proteins were isolated by lysing cells with a lysis buffer containing 20 mM HEPES, 1 mM phenylmethanesulfonyl fluoride, 10 mM sodium fluoride, and a complete mini EDTA-free protease inhibitor cocktail at pH 7.4. Protein concentrations were measured using the Bradford method (27). Extracted proteins were boiled at 95°C for 5 min in sodium dodecyl sulfate buffer, loaded into separate lanes of 7.5 or 12% Mini-PROTEAN TGX gels, and electrophoretically separated. Separated proteins were transferred to polyvinylidene difluoride membranes. The membranes were treated with Block Ace for 50 min at room temperature and incubated with primary antibodies [COX-2 (1:1,000), COX-1 (1:100), p-p65 (1:1,000), t-p65 (1:1,000), p-p105 (1:1,000), t-p105 (1:1,000), t-IκBα (1:1,000), p-ERK1/2 (1:1,000), t-ERK1/2 (1:1,000), p-p38 (1:1,000), t-p38 (1:1,000), p-JNK (1:1,000), t-JNK (1:1,000), and *β*-actin (1:10,000)] for 2 h at room temperature. After washing, the membranes were incubated with horseradish peroxidase-conjugated anti-rabbit or anti-mouse IgG antibody (1:10,000) for 90 min at room temperature. The ECL Western Blotting Analysis System was used to detect immunoreactivity between the antibodies and blots. Chemiluminescent signals from the membranes were detected using the ImageQuant LAS 4000 mini system.

#### Immunocytochemistry

Immunocytochemistry was performed as previously described (10). Synovial fibroblasts were seeded at a density of 3 × 10^5^ cells/ml culture medium into a 35-mm glass bottom dish (Iwaki, Tokyo, Japan) treated with IL-1*β*. The cells were fixed with 4% paraformaldehyde (Nacalai Tesque Inc., Kyoto, Japan) for 15 min and processed for immunocytochemistry to examine the intracellular localization of t-p65. The fixed cells were permeabilized by incubation with 0.2% Triton X-100 (Sigma-Aldrich Inc.) for 15 min at room temperature. Non-specific antibody reactions were blocked for 30 min with Block Ace (DS Pharma Biomedical, Osaka, Japan). The cells were then incubated for 90 min at room temperature with anti-t-p65 rabbit antibody [1:200]. After the cells were washed with PBS containing 0.2% polyoxyethylene (20) sorbitan monolaurate, they were incubated and visualized with Alexa Fluor 594-conjugated F(ab′)2 fragments of goat anti-rabbit IgG (H + L) [1:1,000] and TO-PRO-3-iodide [1:1,000] for 60 min in the dark at 25°C. These samples were washed thrice with PBS containing 0.2% polyoxyethylene (20) sorbitan monolaurate, dried, mounted with ProLong Gold Antifade Reagent, and visualized using a confocal laser scanning microscope (LSM-510; Carl Zeiss AG, Oberkochen, Germany). Co-localization analysis was performed using ZEN software (Carl Zeiss AG).

#### Immunoprecipitation

Total cell lysates (50 µg) were precleared with protein A/G plus Sepharose before incubation with specific antibodies, followed by addition of protein A/G plus Sepharose (10). The total cell lysate was incubated with 20 µg anti-p-p65 or p-pERK1/2 antibody at 4°C for 18 h. The precipitated proteins were dissolved and boiled at 95°C for 5 min in SDS buffer before electrophoresis. Finally, the precipitated proteins were analyzed by western blotting.

### Prostaglandin E2 Assay

Synovial fibroblasts were seeded at a density of 3 × 10^5^ cells/well in 6-well culture plates. The cells were stimulated with canine recombinant IL-1*β* after starvation for 24 h and culture supernatants were collected. To measure culture supernatant prostaglandin E_2_ concentrations, we used an enzyme-linked immunosorbent assay kit according to the kit instructions.

### siRNA Transfection

Canine synovial fibroblasts, seeded at a density of 1 × 10^5^ cells per 35 mm dish or 5 × 10^5^ cells per 90 mm dish, were transfected using Opti-MEM containing 5 μl per ml of Lipofectamine 2000 and 400 nM of p65, p105, ERK1, ERK2, or scramble siRNAs for 6 h (9, 26). **Table 2** lists siRNA sequences. siRNA efficiency was tested using western blotting with antibodies against t-p65 (1:1,000), t-p105 antibody (1:1,000), and t-ERK1/2 (1:1000).

**Table 2:** Sequences for siRNA transfection.

Gene Name	Gene bank ID	siRNA sequences
*p65*	XM_014121307.2	GCAUCUCCCUGGUCACCAA
*p105*	AB183419.1	CUGCAAAGGUUAUUGUUCA
*ERK1*	NM_001252035.1	CCAATGTGCTCCACCGGGA
*ERK2*	NM_001110800.1	CCCAAATGCTGACTCGAAA

### Statistical Analysis

The data from all experiments have been presented as mean ± standard error of measurement. Statistical analyses were performed using StatMate IV. Data from immunoprecipitation and time-course studies were analyzed using the two-tailed Student’s *t*-test and two-way analysis of variance, respectively. Data from other experiments were analyzed using one-way analysis of variance. Tukey’s test was used during *post-hoc* analysis. *P*-values less than 0.05 were considered statistically significant.

## Results

### IL-1*β*-Induced Secretion of Prostaglandin E_2_ *via* Upregulation of COX-2 in Synovial Fibroblasts

**Figure 1:**
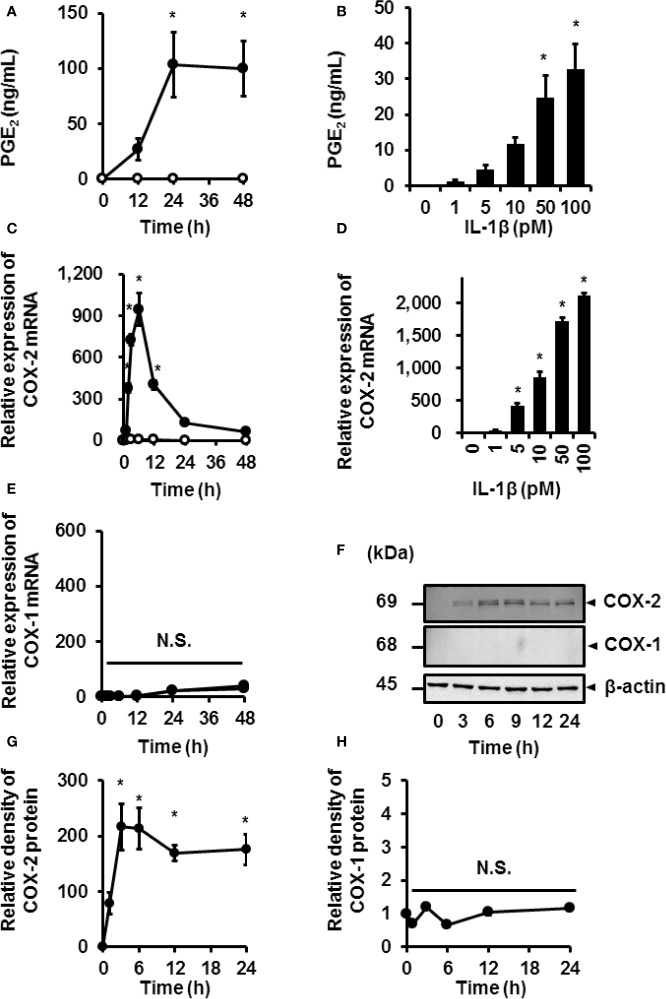
IL-1*β*-induced prostaglandin E_2_ release and COX-2 mRNA and protein levels in canine synovial fibroblasts. Cells treated with (closed circle) or without (open circle) canine recombinant IL-1*β* (100 pM) showed an increase in prostaglandin E_2_ (PGE_2_) release **(A)** and COX-2 mRNA levels **(C)** in a time-dependent manner, but did not affect COX-1 mRNA levels **(E)**. Cells were treated with the indicated concentrations of IL-1*β* for 48 h **(B)** or 6 h **(D)** and assayed for PGE_2_ release **(B)** and COX-2 mRNA levels **(D)** in a dose-dependent manner. Cells treated with IL-1*β* (100 pM) for 0–48 h showed increased protein levels of COX-2 in a time-dependent manner (**F**; first row), but did not affect the protein levels of COX-1 (**F**; second row). The expression of COX-2 **(G)** and COX-1 **(H)** in IL-1*β*-stimulated cells were compared with the expression at 0 h. Results have been represented as mean ± standard error (SE) from biological triplicates. *P < 0.05. Cell lysates (10 μg protein) were used for immunoblotting. *β*-actin was used as the internal standard (**F**; third row).

We investigated the effect of IL-1*β* on the release of prostaglandin E_2_ and expression of COX in canine synovial fibroblasts. IL-1*β* (100 pM) induced the time-dependent release of prostaglandin E_2_ in synovial fibroblasts (**Figure 1A**). Secretion of prostaglandin E_2_ increased with increasing concentrations of IL-1*β* (**Figure 1B**). Constitutive and inducible isoforms of COX (COX-1 and COX-2, respectively) are rate-limiting enzymes that are involved in the synthesis of prostaglandin E_2_ from arachidonic acid (28, 29). IL-1*β* induced COX-2 mRNA expression in a time- (**Figure 1C**) and dose-dependent manner (**Figure 1D**), but had no effect on the expression of COX-1 (**Figure 1E**). The protein levels of COX-2 increased in IL-1*β*-treated synovial fibroblasts (**Figures 1F, G**), but those of COX-1 remained unchanged (**Figures 1F, H**). Taken together, IL-1*β* stimulated the secretion of prostaglandin E_2_ *via* upregulated COX-2 expression in canine synovial fibroblasts.

### Role of Canonical NF-*κ*B Signaling in IL-1*β*-Induced Expression of COX-2

**Figure 2:**
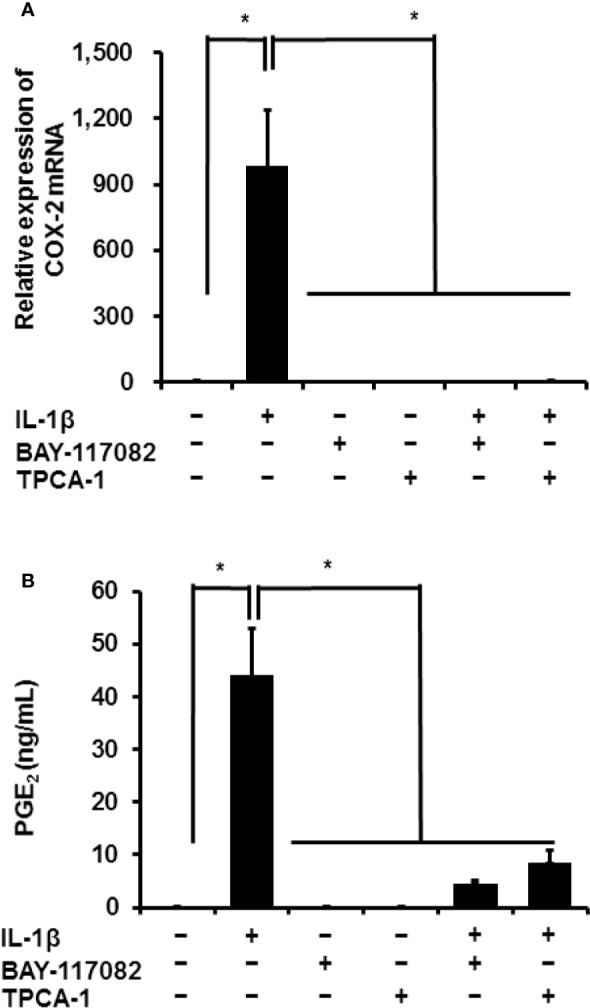
Effect of NF-*κ*B inhibitors on IL-1*β*-induced COX-2 expression. Synovial fibroblasts were pretreated with or without the NF-κB inhibitors BAY11-7082 (10 μM) and TPCA-1 (10 µM), for 1 h and subsequently stimulated with IL-1β (100 pM). After stimulation for 6 or 48 h, we analyzed COX-2 mRNA levels **(A)** and prostaglandin E_2_ release **(B)**. The inhibitors decreased IL-1β-induced COX-2 expression and prostaglandin E_2_ release. Results have been represented as mean ± SE from biological triplicates. *P < 0.05.

Next, we used NF-*κ*B inhibitors to determine the function of canonical NF-*κ*B signaling in IL-1*β*-mediated prostaglandin E_2_ release and COX-2 mRNA levels in canine synovial fibroblasts. Cells were pretreated with NF-*κ*B inhibitors (10 µM BAY11-7082 or 10 µM TPCA-1) for 1 h and subsequently stimulated with IL-1*β* (100 pM) for 48 h. The NF-*κ*B inhibitors significantly reduced the mRNA levels of COX-2 (**Figure 2A**) and IL-1*β*-mediated release of prostaglandin E_2_ (**Figure 2B**). These results suggest that NF-*κ*B signaling is involved in the expression of COX-2 and subsequent IL-1*β*-mediated release of prostaglandin E_2_.

**Figure 3:**
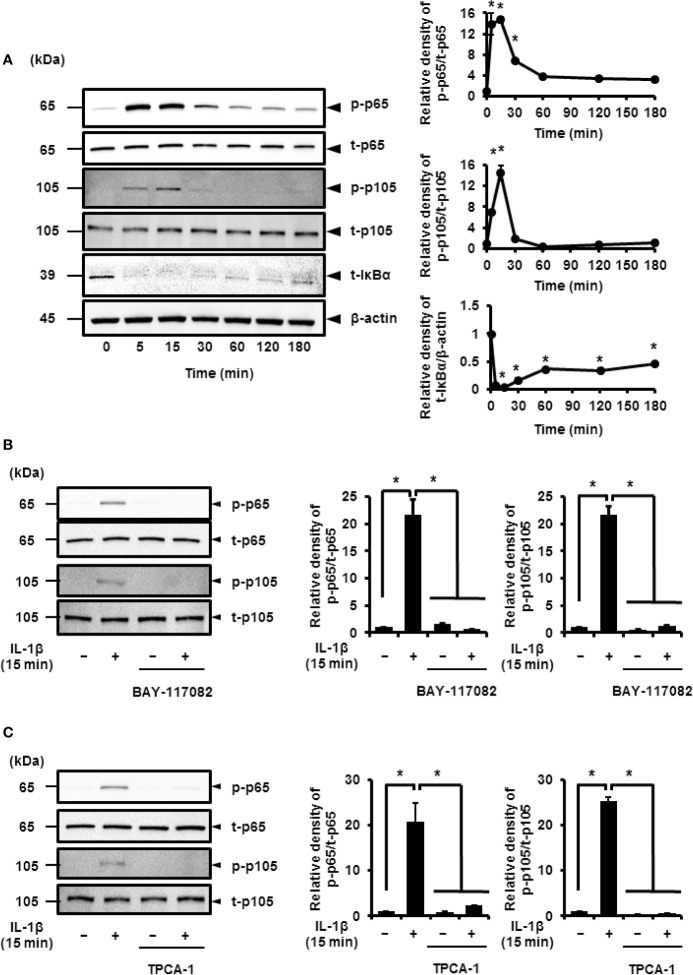
IL-1*β-*induced activation of NF-*κ*B signaling. **(A)** Western blotting for, the levels of phosphorylated p65 (p-p65), total p65 (t-p65), phosphorylated p105 (p-p105), total p105 (t-p105), and total IκBα (t-IκBα) in cells treated with IL-1β (100 pM). Relative expression of p-p65, p-p105, and t-IκBα as compared to the levels at 0 h (right panel). **(B, C)** Cells were pretreated with or without the NF-κB inhibitors BAY11-7082 (10 μM; **B**) and TPCA-1 (10 µM; **C**) for 1 h followed by stimulation with IL-1β for 15 min. The inhibitors reduced IL-1β-induced phosphorylation of p65 and p105. Relative levels of p-p65 and p-p105 compared with those in the absence of IL-1β (right panel). Results have been represented as mean ± SE from biological triplicates. *P < 0.05. For the immunoblotting, cell lysates (10 μg protein) were used for immunoblotting. *β*-actin was used as the internal standard **(A)**.

Pro-inflammatory cytokines, such as IL-1*β*, stimulate the phosphorylation of p65 and p105 subunits, which activate the transcriptional function of NF-*κ*B (30–35). Thus, cells treated with IL-1*β* showed transient phosphorylation of p65 and p105 that reached peak levels at 15 min (**Figure 3A**). I*κ*B*α* was found to degrade in IL-1*β*-treated cells in a time-dependent manner (**Figure 3A**). These observations strongly suggest that IL-1*β* activates NF-*κ*B signaling.

**Figure 4:**
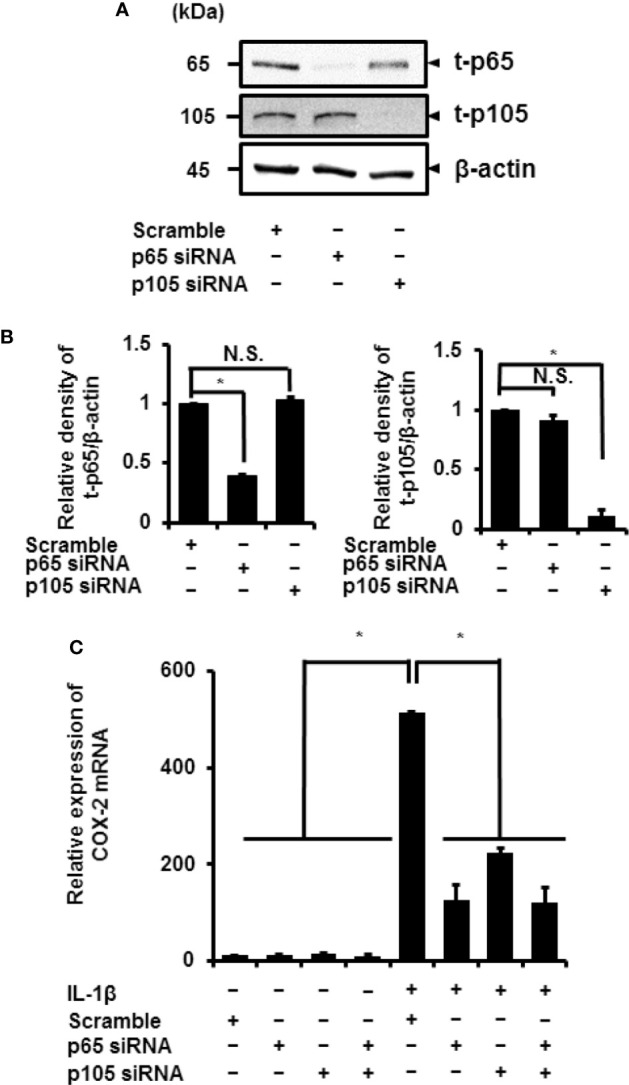
Inhibition of IL-1*β*-induced COX-2 expression in synovial fibroblasts depleted of p65 and p105. **(A)** Protein levels of total p65 (t-p65), total p105 (t-p105), and *β*-actin (internal standard) were detected by immunoblotting in cells transfected with p65, p105, or scramble siRNAs (control). **(B)** The relative levels of t-p65 and t-p105 as compared to the levels in the control samples. **(C)** Cells transfected with p65, p105, or scramble siRNAs were stimulated with IL-1*β* for 6 h. Depleting cells of p65 and p105 inhibited IL-1*β*-induced COX-2 expression. Results have been represented as mean ± SE from biological triplicates. *P < 0.05. Cell lysates (10 μg protein) were used for immunoblotting.

Cells pretreated with NF-*κ*B inhibitors BAY11-7082 or TPCA-1 for 1 h were stimulated using IL-1*β* for 15 min. Subsequently, we found a significant decrease in the IL-1*β*-mediated phosphorylation of p65 and p105 (**Figures 3B, C**). Then, we determined the role of p65 and p105 in IL-1*β*-mediated expression of COX-2. The protein levels of total p65 (t-p65) or p105 (t-p105) decreased significantly in cells transfected with siRNAs targeting p65 or p105 as compared to those in cells transfected with scramble siRNA (**Figures 4A, B**). We also observed that transfection of p65 or p105 siRNA had no effect of the expression of t-p105 or t-p65, respectively (**Figures 4A, B**). Cells depleted of p65 or p105 showed a reduction in IL-1*β*-induced COX-2 mRNA levels (**Figure 4C**); COX-2 mRNA levels did not significantly differ between cells depleted of p65 and p105. Thus, p65 and p105 play a role in IL-1*β*-induced expression of COX-2 in synovial fibroblasts.

### Role of ERK1/2 Signaling in IL-1*β*-Induced Expression of COX-2

**Figure 5:**
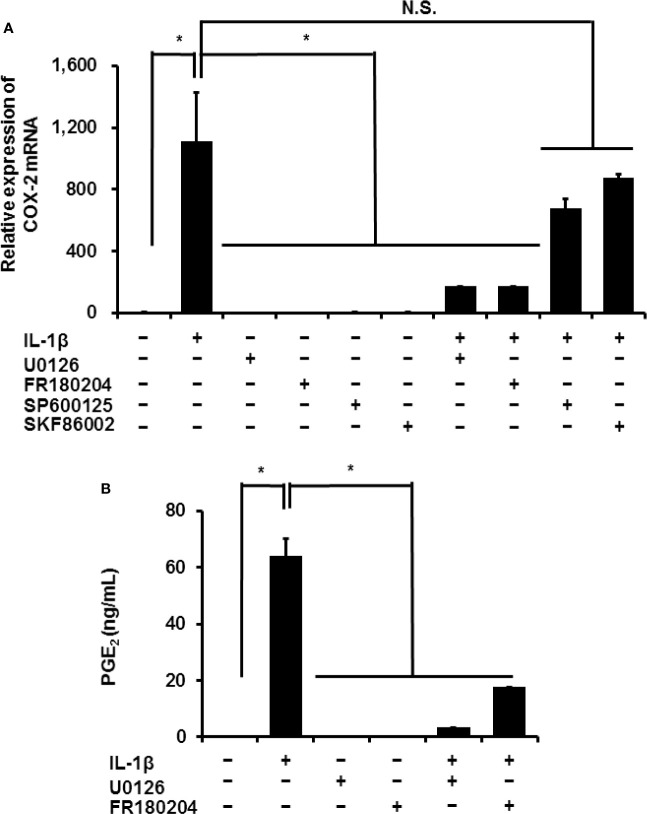
Effect of MAPK inhibitors on IL-1*β*-induced COX-2 expression. Synovial fibroblasts were pretreated with or without the MEK inhibitor U0126 (20 µM), ERK1/2 inhibitor FR180204 (50 µM), JNK inhibitor SP600125 (10 µM), and p38 inhibitor SKF86002 (10 µM) for 1 h and subsequently stimulated with IL-1*β*. After stimulation for 6 h or 48 h, we determined COX-2 expression **(A)** and prostaglandin E_2_ release **(B)**. IL-1*β*-induced COX-2 expression and prostaglandin E_2_ release were significantly reduced by the inhibitors. Results have been represented as mean ± SE from biological triplicates. *P < 0.05.

In mammalian cells, MAPK signaling is important in inflammation. Three MAPK signaling pathways have been characterized: ERK1/2, JNK, and p38 MAPK (36, 37). We investigated the contribution of MAPK signaling in the IL-1*β*-induced expression of COX-2 in canine synovial fibroblasts. Inhibitors of ERK1/2 and its upstream regulator MEK, FR180204 (50 μM) and U0126 (20 μM), respectively, decreased the IL-1*β*-induced expression of COX-2 (**Figure 5A**). However, inhibitors of JNK and p38, SP600125 (10 μM) and SKF86002 (10 μM), respectively did not significantly alter COX-2 mRNA levels. Inhibition of MEK or ERK reduced the IL-1*β*-induced release of prostaglandin E_2_ (**Figure 5B**).

**Figure 6:**
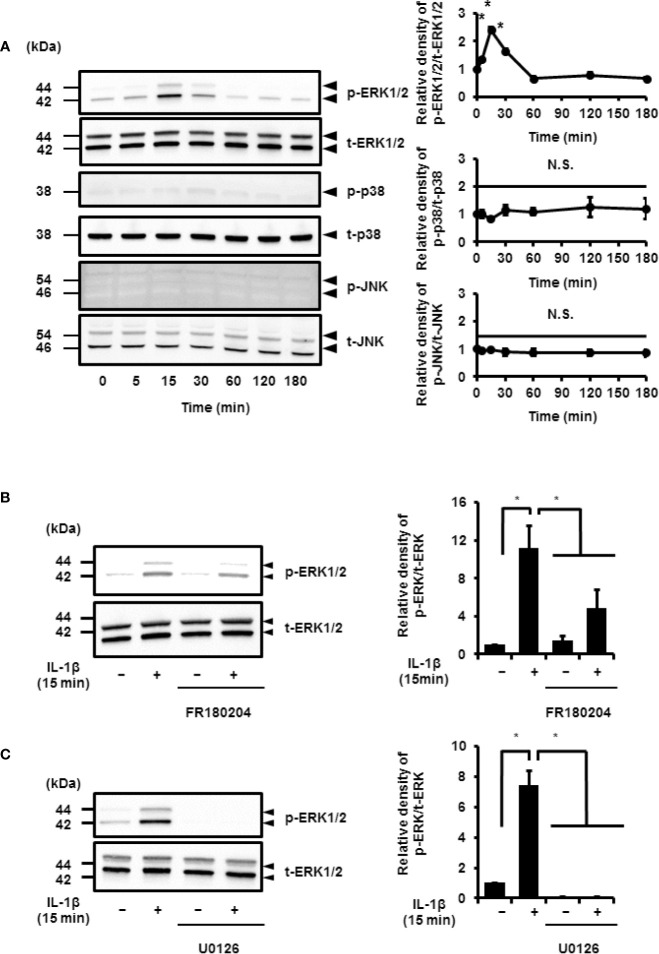
IL-1*β*-induced activation of ERK1/2 signaling. **(A)** Western blotting for the levels of phosphorylated ERK1/2 (p-ERK1/2), total ERK1/2 (t-ERK1/2), phosphorylated JNK (p-JNK), total JNK (t-JNK), phosphorylated p38 MAPK (p-p38), and total p38 MAPK (t-p38) in cells treated with IL-1*β* (100 pM). Relative levels of p-ERK1/2, p-JNK, and p-p38 compared to the levels at 0 h (right panel). **(B, C)** Cell were pretreated with the ERK1/2 inhibitor FR180204 (50 µM; **B**) and MEK inhibitor U0126 (20 µM; C) for 1 h and stimulated with IL-1*β* for 15 min. MEK and ERK1/2 inhibitors significant reduced IL-1*β*-induced phosphorylation of ERK1/2. Relative levels of p-ERK1/2 as compared to those without IL-1*β* (right panel). Results have been represented as mean ± SE from biological triplicates. *P < 0.05. Cell lysates (10 μg protein) were used for immunoblotting. *β*-actin was used as an internal standard **(A)**.

**Figure 7:**
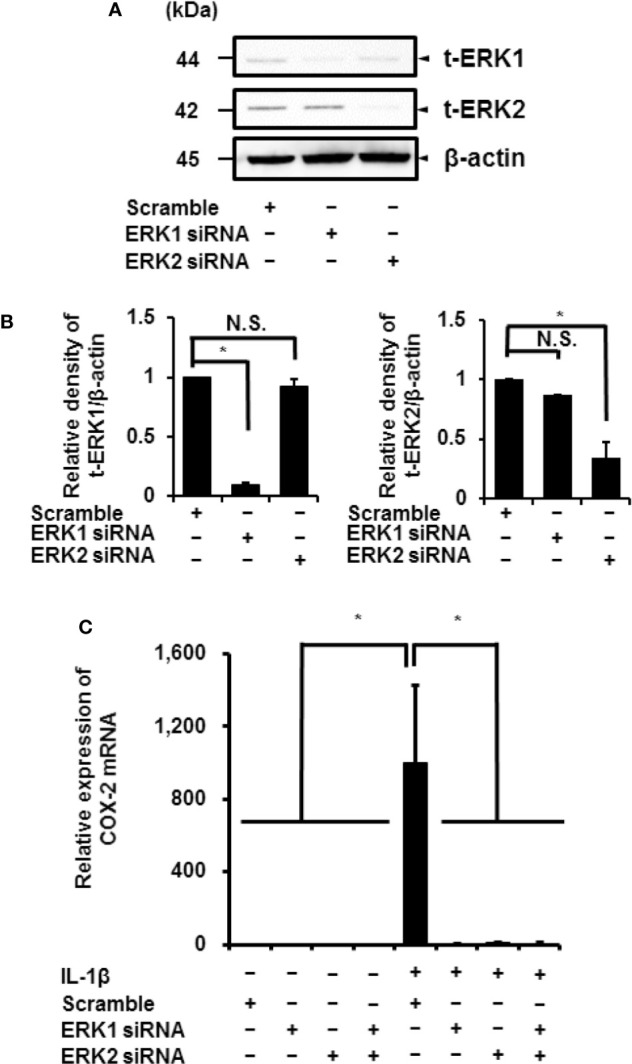
Inhibition of IL-1*β*-induced COX-2 expression in synovial fibroblasts transfected with ERK1 and ERK2 siRNAs. **(A)** Protein levels of total ERK1 (t-ERK1), total ERK2 (t-ERK2), and *β*-actin (an internal standard) were detected by immunoblotting in cells transfected with ERK1, ERK2, or scramble siRNAs (control). Cell lysates (10 μg protein) were used for immunoblotting. **(B)** The relative levels of t-ERK1 and t-ERK2 as compared to the levels in the control cells. **(C)** ERK1 and ERK2 depletion decreased IL-1*β*-induced COX-2 expression. Results have been represented as mean ± SE from biological triplicates. *P < 0.05.

IL-1*β* stimulated the phosphorylation of ERK1/2 in a time-dependent manner. However, the phosphorylation levels of p38 MAPK and JNK remained unchanged. Phosphorylation reached a peak 15 min after IL-1*β* stimulation (**Figure 6A**). Cells pretreated with MEK and ERK inhibitors showed a reduction in IL-1*β*-induced phosphorylation of ERK1/2 (**Figures 6B, C**). These results suggest that MEK/ERK1/2 signaling axes are dominantly involved in the IL-1*β*-induced expression of COX-2 in canine synovial fibroblasts. To confirm the role of ERK1/2 in IL-1*β*-induced COX-2 expression, we used siRNAs against ERK1 or ERK2 in the fibroblasts. We observed significant decreases in the ERK1 or ERK2 protein levels as compared to cells transfected with scramble siRNA (**Figures 7A, B**). We also observed that transfection of ERK1 or ERK2 siRNA had no effect of the expression of t-ERK2 or t-ERK1, respectively (**Figures 7A, B**). IL-1*β*-induced expression of COX-2 was attenuated in cells transfected with siRNAs for ERK1 or ERK2 as compared to mRNA levels of COX-2 in scramble siRNA-transfected cells (**Figure 7C**). The extent of reduction in the mRNA levels of IL-1*β*-induced COX-2 was similar in cells transfected with siRNAs targeting either ERK1 or ERK2 (**Figure 7C**). Taken together, our data indicate that ERK1/2 signaling is involved in the IL-1*β*-induced expression of COX-2 in canine synovial fibroblasts.

### Inhibition of IL-1*β*-Induced ERK1/2 Activation Using NF-*κ*B Inhibitors

**Figure 8:**
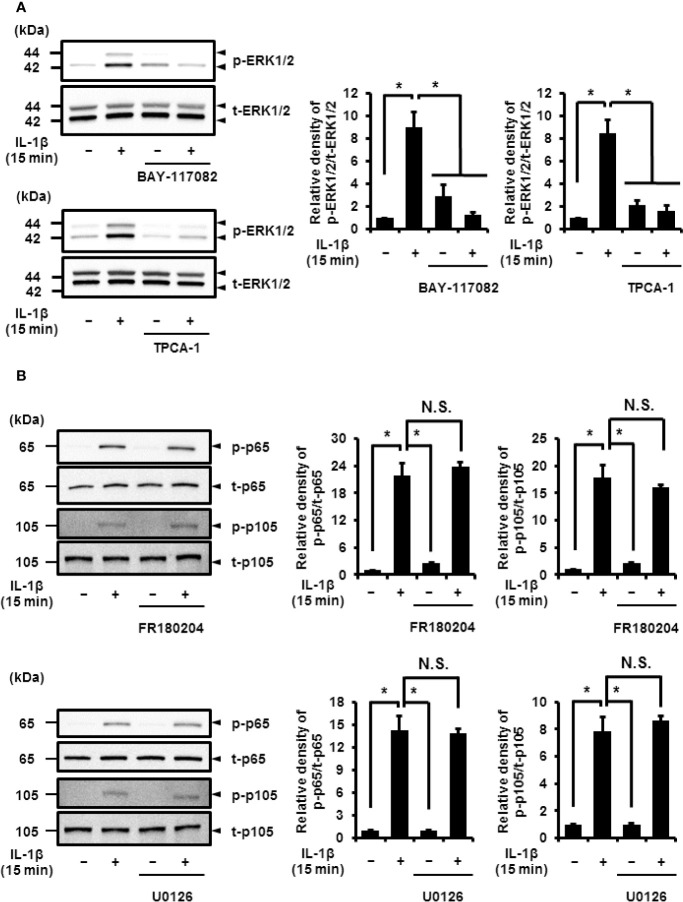
Effect of NF-*κ*B inhibitors on IL-1*β*-induced phosphorylation of ERK1/2. **(A)** Cells were pretreated with the NF-***κ***B inhibitors BAY11-7082 (10 μM) and TPCA-1 (10 µM) for 1 h and stimulated with IL-1*β* for 15 min. NF-κB inhibitors significantly reduced IL-1*β*-induced phosphorylation of ERK1/2 as compared to phosphorylation in the absence of IL-1*β*. Representative blots and relative levels of p- and t-ERK1/2 have been illustrated in the left and right panels, respectively. **(B, C)** Cells were pretreated with the ERK1/2 inhibitor FR180204 (50 µM; **B**) and MEK inhibitor U0126 (20 µM; C) for 1 h followed by stimulation with IL-1*β* for 15 min. ERK1/2 and MEK inhibitors had no effect on IL-1*β*-induced phosphorylation of p65 and p105. Cell lysates (10 μg protein) were used for immunoblotting. Representative blots and relative levels of p- and t-p65 and p105 have been shown in the left and right panels, respectively. (Right) Results have been represented as mean ± SE from biological triplicates. *P < 0.05.

Next, we determined the relationship between NF-*κ*B and MEK/ERK signaling. In cells pretreated with NF-*κ*B inhibitors, BAY 11-7082 and TPCA-1, IL-1*β* failed to induce the phosphorylation of ERK1/2, but had no effect on the total protein levels of ERK1/2 (**Figure 8A**). However, the use of MEK and ERK1/2 inhibitors, U0126 and FR180204, respectively, had no effect on IL-1*β*-induced phosphorylation of p65 and p105 (**Figure 8B**). These results suggest that the activation of NF-*κ*B is pivotal for ERK1/2 signaling.

### The Non-Transcriptional and Translational Function of Canonical NF-*κ*B Regulates IL-1*β*-Induced Activation of ERK1/2

**Figure 9:**
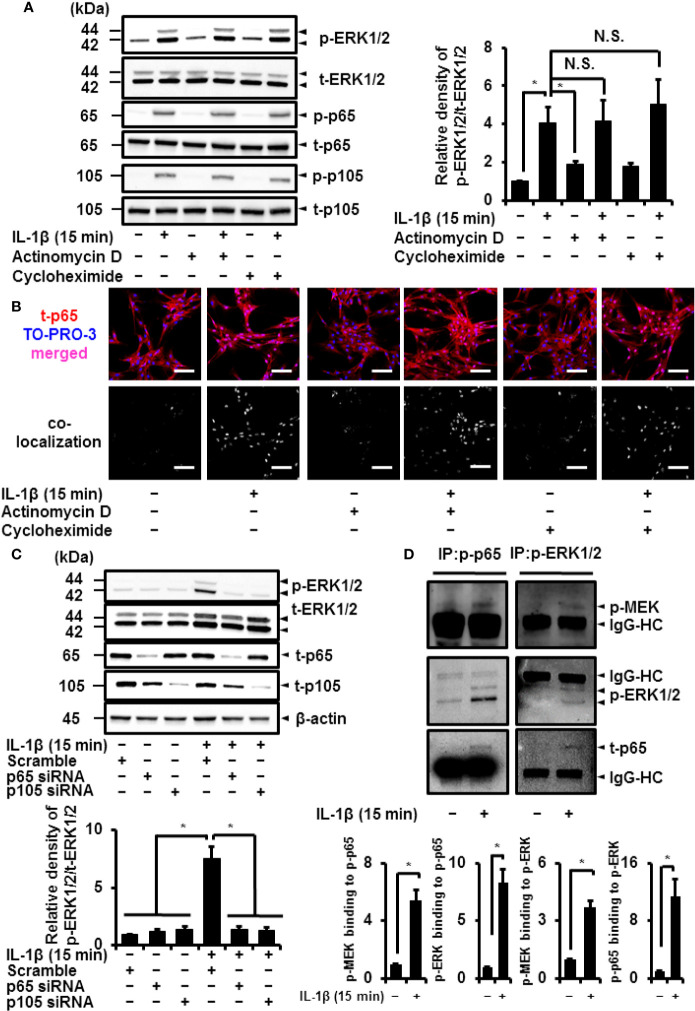
Canonical NF-*κ*B signaling non-transcriptionally and non-translationally regulates IL-1*β*-induced ERK1/2 activation. **(A)** Cells were pretreated with the transcription inhibitor actinomycin D (Act D; 1 μM) and translation inhibitor cycloheximide (CHX; 100 μM) for 6 h and stimulated with IL-1*β* for 15 min. ActD and CHX had no effect on IL-1*β*-induced phosphorylation of ERK1/2, p65 and p105. Representative blots and relative levels of p- and t-ERK1/2, p65 and p105 have been illustrated in the left and right panels, respectively. **(B)** Cells were pretreated with the transcription inhibitor actinomycin D (Act D; 1 μM) and translation inhibitor cycloheximide (CHX; 100 μM) for 6 h and stimulated with IL-1*β* for 15 min. Subsequently, immunocytochemistry and co-localization analysis with nuclear staining dye TO-PRO-3 were performed. The nuclear localization of t-p65 was detected in the cells treated with IL-1*β*, whereas t-p65 showed cytoplasmic localization in control cells. IL-1*β* induced the nuclear localization of t-p65 in the presence of actinomycin D and cycloheximide. **(C)** Western blotting for the levels of p-ERK1/2, t-ERK1/2, t-p65 and t-p105 in cells transfected p65, p105, or scramble siRNAs. Decreased IL-1*β*-induced phosphorylation of ERK1/2 was observed in p65 or p105 siRNA-transfected cells. Representative blots for the levels of p-ERK1/2, t-ERK1/2, t-p65, and t-p105 and relative expression of p-ERK1/2 have been compared with the levels in cells transfected with scramble siRNA in the left and right panels, respectively. Results have been represented as mean ± SE from biological triplicates. *P < 0.05. Cell lysates (10 μg protein) were used for immunoblotting. *β*-actin was used as an internal standard **(C)**. **(D)** Western blotting for the levels of p-MEK, p-ERK1/2 and p-p65 in the fraction precipitated with p-p65 and p-ERK1/2. Results have been represented as mean ± SE from biological triplicates. *P < 0.05. Cell lysates (50 μg protein) were used for co-immunoprecipitation experiments. IgG-heavy chain (IgG-HC) was used as an internal standard **(D)**.

NF-*κ*B plays a crucial role in transcriptionally and translationally regulating immune and inflammatory responses (5, 6). We determined the contribution of the transcriptional or translational function of NF-*κ*B in IL-1*β*-induced phosphorylation of ERK1/2. The transcription inhibitor actinomycin D (1 μM) and translation inhibitor cycloheximide (100 μM) had no effect on IL-1*β*-induced activation of p65 and p105 (**Figure 9A**). Actinomycin D and cycloheximide also failed to attenuate IL-1*β*-induced phosphorylation of ERK1/2 (**Figure 9A**), suggesting that ERK1/2 phosphorylation by IL-1*β* is independent of transcriptional and translational regulation. It has been reported that the function of NF-*κ*B was regulated by the nuclear localization. Then, we examined the cellular localization of NF-*κ*B in IL-1*β*-treated cells by immunocytochemistry and co-localization analysis with a nucleic acid staining dye TO-PRO-3. The nuclear localization of p65 was observed in the cells treated with IL-1*β*, whereas p65 showed cytoplasmic localization in control cells (**Figure 9B**). IL-1*β* induced the nuclear localization of p65 in the presence of actinomycin D and cycloheximide (**Figure 9B**), suggesting that NF-*κ*B acts as a transcription factor even in the presence of the inhibitors for transcription and translation.

To confirm the importance of NF-*κ*B in the activation of ERK1/2, we depleted cells of p65 or p105 to observe a reduction in IL-1*β*-induced phosphorylation of ERK1/2 (**Figure 9C**). Then, we investigated the mechanism of the interaction between activated NF-*κ*B and phosphorylated ERK1/2. As shown in **Figure 9D**, we detected that the levels of phosphorylated p65 or ERK1/2 in the fraction precipitated with anti-phosphorylated p65 or anti-phosphorylated ERK1/2, respectively, were increased. We observed that the levels of phosphorylated ERK1/2 in the fraction precipitated with anti-phosphorylated p65 increased in the cells treated with IL-1*β*. The increase in phosphorylated p65 in the fraction precipitated with anti-phosphorylated ERK1/2 was also detected (**Figure 9D**). Regarding the density of the IgG heavy chain (HC) band, there is no significant difference between control and the IL-1*β*-treated cells. These results suggest that the complex formation of activated NF-*κ*B and phosphorylated ERK1/2 is induced by IL-1*β*. We further investigated whether phosphorylated p65 and phosphorylated ERK1/2 form a complex with phosphorylated MEK as an upstream regulator of ERK1/2. As shown in **Figure 9D**, we observed that the levels of phosphorylated MEK increased in the fraction precipitated with anti-phosphorylated p65 and ERK1/2, suggesting that phosphorylated MEK also forms a complex with NF-*κ*B and ERK1/2 in the cells treated with IL-1*β*.

**Figure 10:**
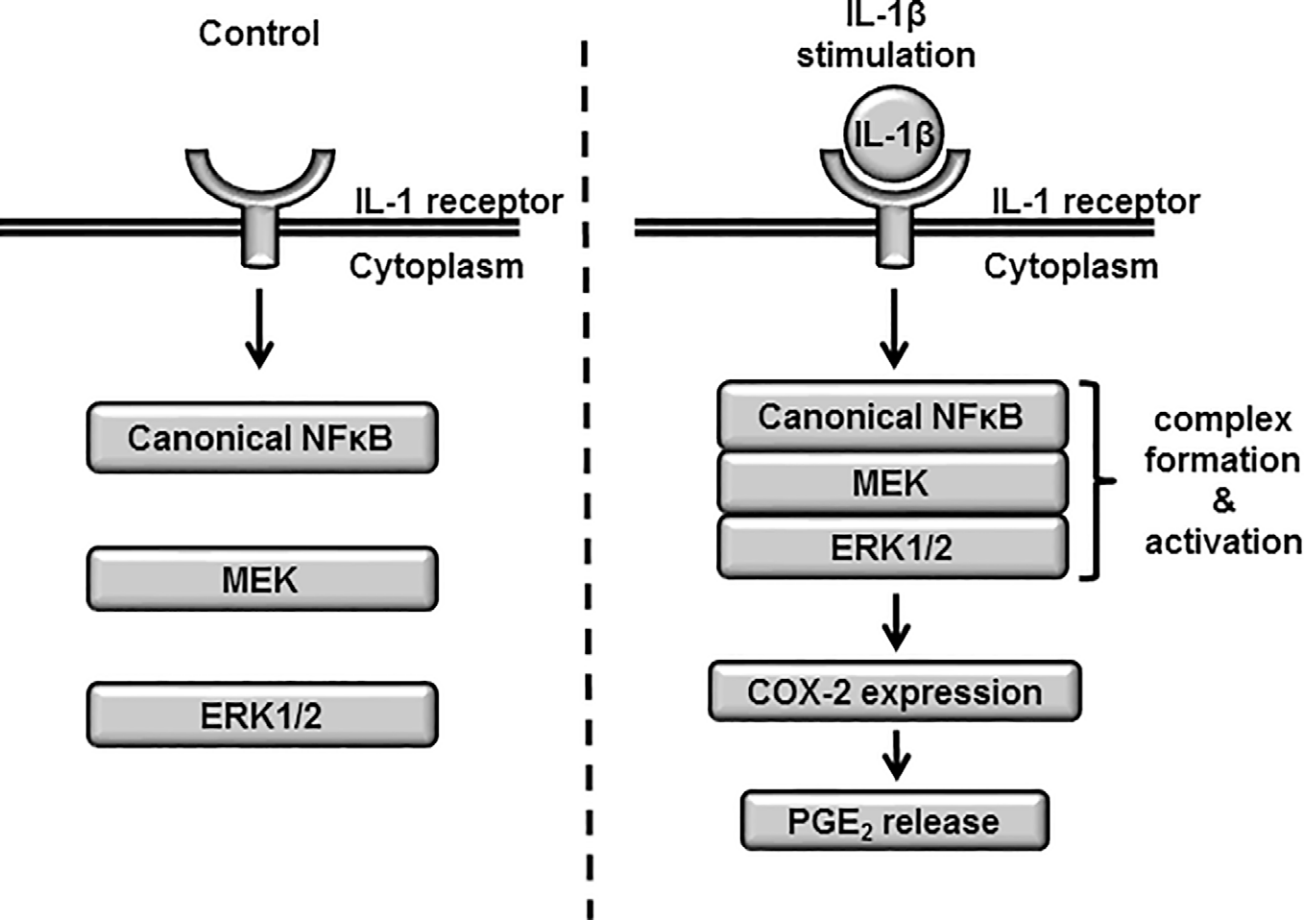
The proposed model highlighting atypical role of NF-*κ*B signaling in the ERK1/2 regulation *via* the complex formation of NF-*κ*B/MEK/ERK in IL-1*β*-treated synovial fibroblasts. When the cells were stimulated with IL-1*β*, the complex formation of NF-*κ*B/MEK/ERK was induced, which promoted the activation of ERK1/2, the expression of COX-2 and prostaglandin E_2_ synthesis.

Based on these results, we propose a model of atypical role of NF-*κ*B signaling in the ERK1/2 regulation *via* the complex formation of NF-*κ*B/MEK/ERK (**Figure 10**).

## Discussion

In this study, we have demonstrated that pro-inflammatory cytokine IL-1*β*-induced expression of COX-2 mediated the release of prostaglandin E_2_ in canine synovial fibroblasts. COX-2 expression is induced by stimuli, such as IL-1*β* and TNF-α, in the synovium of patients with RA, thereby enhancing the production of prostanoids, including prostaglandin E_2_, that are involved in inflammation. Synovial prostaglandin E_2_ results in the manifestation of various pathological symptoms, including bone/cartilage degradation, inhibition of matrix synthesis, general discomfort, and body weight loss (38, 39). These studies suggest that the expression of COX-2 in the synovium plays a crucial role in the pathogenesis of RA *via* the synthesis of prostaglandins.

The NF-*κ*B/p65/p105 axis was involved in IL-1*β*-mediated expression of COX-2 in synovial fibroblasts. NF-*κ*B is a transcription factor important for the regulation of a wide range of target genes involved in physiological and pathological processes. The NF-*κ*B family of proteins comprises five members: p65 (RelA), RelB, c-Rel, p50, and p52, that interact with each other to form homo- or heterodimers with distinct functions. Furthermore, NF-*κ*B signaling can be classified into canonical and noncanonical signaling (40). In the canonical pathway, formation of the p50/p65 heterodimer is a key step in the activation of NF-*κ*B (41, 42). In resting cells, p50 and p65 form an inactive complex with the inhibitory protein I*κ*B*α*. I*κ*B kinase is activated by exogenous signals, such as IL-1*β*, that phosphorylates I*κ*B*α*, thereby inducing its ubiquitination and proteasomal degradation. The p50/p65 complex that dissociates from I*κ*B*α* is transcriptionally active and induces the expression of the target genes of NF-*κ*B (40). In this study, selective inhibitors of NF-*κ*B, BAY11-7082, and TPCA-1 reduced IL-1*β*-mediated COX-2 expression. The expression of COX-2 decreased significantly in cells depleted of p65 and p105 (the precursor of p50). Thus, the activation of canonical NF-*κ*B signaling is important for IL-1*β*-induced expression of COX-2 in canine synovial fibroblasts.

Multiple MAPK signaling pathways coordinate and integrate cellular response to diverse stimuli, such as cytokines like IL-1*β*. However, the activation of MAPK signaling is highly dependent on cellular context. In this study, we have demonstrated that pharmacological inhibition of MEK and ERK1/2 drastically reduced IL-1*β*-induced COX-2 expression and prostaglandin E_2_ release; IL-1*β* induced the activation of MEK/ERK1/2. Taken together, these results suggest that the activation of MEK/ERK1/2 signaling plays a crucial role in COX-2 expression and prostaglandin E_2_ release in synovial fibroblasts that is important in the pathogenesis of synovitis in patients with RA.

Activation of MAPK signaling stimulates NF-*κ*B that induces transcriptional regulation (43). ERK1/2 activates canonical NF-*κ*B signaling in rat vascular smooth muscle cells that regulates IL-1*β*-induced gene expression (18). Canonical NF-*κ*B signaling is controlled by ERK1/2 in other cells, *e.g*., the human monocyte THP-1 cell line (20). Therefore, we investigated the interaction between canonical NF-*κ*B signaling and ERK1/2 activation. Surprisingly, we found that U0126 and FR180204, inhibitors of MEK and ERK1/2, respectively, had no effect on the activation of canonical NF-*κ*B signaling induced by IL-1*β*. In contrast, NF-*κ*B inhibitors BAY 11-7082 and TPCA-1 inhibited IL-1*β*-induced ERK1/2 phosphorylation. IL-1*β*-induced ERK1/2 phosphorylation was inhibited in cells depleted of p65 and p105. Furthermore, the pharmacological inhibitors for transcription and translation, actinomycin D and cycloheximide, respectively, failed to inhibit IL-1*β*-induced ERK1/2 phosphorylation. These observations collectively suggest that the non-transcriptional/translational function of canonical NF-*κ*B regulates the activation of ERK1/2 signaling in canine synovial fibroblasts.

The activation of ERK1/2 signaling by canonical NF-*κ*B in canine synovial fibroblasts remains to be understood. In this study, we observed that p65 and p105 phosphorylation was an indicator of NF-*κ*B activation. These changes are attributed to the dissociation of NF-*κ*B from inhibitory I*κ*B*α* and are necessary for nuclear localization, transcriptional activity, and other functions of NF-*κ*B (38). Furthermore, NF-*κ*B interacts with a variety of proteins (44, 45). Tieri et al. constructed the map of the interactome of NF-*κ*B based on protein–protein interaction data from multiple databases and reported the crosstalk between NF-*κ*B and MAPK signaling, supporting an atypical role of canonical NF-*κ*B signaling in ERK1/2 activation (45). In this study, the level of phosphorylated ERK1/2 in the fraction precipitated with anti-phosphorylated p65 increased in the cells treated with IL-1*β*. The increase in phosphorylated p65 in the fraction precipitated with anti-phosphorylated ERK1/2 was also detected. These observations suggest that the complex formation of activated NF-*κ*B and phosphorylated ERK1/2 is induced by IL-1*β*. Since we observed that the MEK inhibitor U0126 attenuated IL-1*β*-induced COX-2 mRNA expression and ERK1/2 phosphorylation, we further examined the interaction of phosphorylated MEK with phosphorylated p65 and phosphorylated ERK1/2. Our co-immunoprecipitation data showed that the increase in phosphorylated MEK in the fraction precipitated with anti-phosphorylated p65 and ERK1/2. These observations collectively suggest that NF-*κ*B signaling regulates the activation of MEK/ERK signaling *via* the complex formation of NF-*κ*B/MEK/ERK. Since the molecular feature of the NF-*κ*B/MEK/ERK complex has been unclear, the detection of other components of NF-*κ*B/MEK/ERK complex is currently underway in our laboratory. In addition, to confirm whether our proposed model is ubiquitously existed, studies targeting the atypical role of NF-*κ*B signaling in the ERK1/2 regulation in several types of cells is indeed our next research subject.

In conclusion, we demonstrated that 1) canonical NF-*κ*B signaling contributes to IL-1*β*-induced COX-2 expression and subsequent prostaglandin E_2_ release in canine synovial fibroblasts, and 2) the activation of canonical NF-*κ*B signaling non-transcriptionally and non-translationally activates ERK1/2 signaling. Based on our study findings, we propose a model highlighting the importance of IL-1*β*-induced NF-*κ*B/ERK1/2/COX-2 signaling in canine synovial fibroblasts (**Figure 9C**). The atypical role of canonical NF-*κ*B signaling in IL-1*β*-induced ERK1/2 activation may serve as promising molecular targets for the development of therapeutics in treating patients with RA and synovitis.

## Data Availability Statement

All datasets presented in this study are included in the article/**Supplementary Material**.

## Author Contributions

RN and HS designed and planned the experiments. RN, TKi, SN, NK, and YS prepared and measured samples. RN, TKi, SN, NK, YS, and TKo analyzed the data. RN, JY, TN, and HS interpreted the data and wrote the paper. All authors contributed to the article and approved the submitted version.

## Funding

This work was supported in part by a Grant-in-Aid for Scientific Research (grant no. 18K14594; RN, grant no. 19K06389; TN) from the Ministry of Education, Science, Sports, and Culture of Japan (https://www.jsps.go.jp/j-grantsinaid/). The funders had no role in the study design, data collection and analysis, decision to publish, or preparation of the manuscript.

## Conflict of Interest

The authors declare that the research was conducted in the absence of any commercial or financial relationships that could be construed as a potential conflict of interest.

## References

[1] Iwakura Y Roles of IL-1 in the development of rheumatoid arthritis: consideration from mouse models. Cytokine Growth Factor Rev (2002) 13:341–55. 10.1016/S1359-6101(02)00021-7 12220548

[2] Ruscitti P Cipriani P Liakouli V Carubbi F Berardicurti O Di Benedetto P The Emerging Role of IL-1 Inhibition in Patients Affected by Rheumatoid Arthritis and Diabetes. Rev Recent Clin Trials (2018) 13:210–4. 10.2174/1574887113666180314102651 29542422

[3] Gabay C Lamacchia C Palmer G IL-1 pathways in inflammation and human diseases. Nat Rev Rheumatol (2010) 6:232–41. 10.1038/nrrheum.2010.4 20177398

[4] Gabay C IL-1 inhibitors: novel agents in the treatment of rheumatoid arthritis. Expert Opin Investig Drugs (2000) 9:113–27. 10.1517/13543784.9.1.113 11060665

[5] Lawrence T The nuclear factor NF-κB pathway in inflammation. Cold Spring Harbor Pespect Biol (2009) 1:a001651. 10.1101/cshperspect.a001651 PMC2882124 20457564

[6] Hayden MS Ghosh S NF-κB, the first quarter-century: remarkable progress and outstanding questions. Genes Dev (2012) 26:203–34. 10.1101/gad.183434.111 PMC3278889 22302935

[7] Tsuchiya H Nakano R Konno T Okabayashi K Narita T Sugiya H Activation of MEK/ERK pathways through NF-κB activation is involved in interleukin-1β-induced cyclooxygenease-2 expression in canine dermal fibroblasts. Vet Immunol Immunopathol (2015) 168:223–32. 10.1016/j.vetimm.2015.10.003 26549149

[8] Kitanaka T Nakano R Kitanaka N Kimura T Okabayashi K Narita T JNK activation is essential for activation of MEK/ERK signaling in IL-1β-induced COX-2 expression in synovial fibroblasts. Sci Rep (2017) 7:39914. 10.1038/srep39914 28054591 PMC5215076

[9] Namba S Nakano R Kitanaka T Kitanaka N Nakayama T Sugiya H ERK2 and JNK1 contribute to TNF-α-induced IL-8 expression in synovial fibroblasts. PloS One (2017) 12:e0182923. 10.1371/journal.pone.0182923 28806729 PMC5555573

[10] Nakano R Kitanaka T Namba S Kitanaka N Sugiya H Protein kinase Cϵ regulates nuclear translocation of extracellular signal-regulated kinase, which contributes to bradykinin-induced cyclooxygenase-2 expression. Sci Rep (2018) 8:8535. 10.1038/s41598-018-26473-7 29867151 PMC5986758

[11] Kitanaka N Nakano R Sugiura K Kitanaka T Namba S Konno T Interleukin-1β promotes interleulin-6 expression via ERK1/2 signaling pathway in canine dermal fibroblasts. PloS One (2019) 14:e0220262. 10.1371/journal.pone.0220262 31344106 PMC6658082

[12] Kitanaka N Nakano R Sakai M Kitanaka T Namba S Konno T ERK1/ATF-2 signaling axis contributes to interleukin-1β-induced MMP-3 expression in dermal fibroblasts. PloS One (2019) 14:e0222869. 10.1371/journal.pone.0222869 31536594 PMC6752866

[13] Nakano R Kitanaka T Namba S Kitanaka N Sato M Shibukawa Y All-trans retinoic acid induces reprogramming of canine dedifferentiated cells into neuron-like cells. PloS One (2020) 15:e0229892. 10.1371/journal.pone.0229892 32231396 PMC7108708

[14] Imajo M Tsuchiya Y Nishida E Regulatory mechanisms and functions of MAP kinase signaling pathways. IUBMB Life (2006) 58:312–7. 10.1080/15216540600746393 16754324

[15] Kim EK Choi EJ Compromised MAPK signaling in human diseases: an update. Arch Toxicol (2015) 89:867–82. 10.1007/s00204-015-1472-2 25690731

[16] Schulze-Osthoff K Ferrari D Riehemann K Wesselborg S Regulation of NF-κB activation by MAP kinase cascades. Immunobiol (1997) 198:35–49. 10.1016/S0171-2985(97)80025-3 9442376

[17] De Smaele E Zazzeroni F Papa S Nguyen DU Jin R Jones J Induction of *gadd*45β by NF-κB downregulates pro-apoptotic JNK signalling. Nature (2001) 414:308–13. 10.1038/35104560 11713530

[18] García-García E Sánchez-Mejorada G Rosales C Phosphatidylinositol 3-kinase and ERK are required for NF-κB activation but not for phagocytosis. J Leukoc Biol (2001) 70:649–58. 10.1189/jlb.70.4.649 11590203

[19] Vermeulen L De Wilde G Van Damme P Vanden Berghe W Haegeman G Transcriptional activation of the NF-κB p65 subunit by mitogen- and stress-activated protein kinase-1 (MSK1). EMBO J (2003) 22:1313–24. 10.1093/emboj/cdg139 PMC151081 12628924

[20] Jiang B Xu S Hou X Pimentel DR Brecher P Cohen RA Temporal control of NF-κB activation by ERK differentially regulates interleukin-1β-induced gene expression. J Biol Chem (2004) 279:1323–9. 10.1074/jbc.M307521200 14581482

[21] Kim JH Na HK Pak YK Lee YS Lee SJ Moon A Roles of ERK and p38 mitogen-activated protein kinases in phorbol ester-induced NF-κB activation and COX-2 expression in human breast epithelial cells. Chem Biol Interact (2008) 171:133–41. 10.1016/j.cbi.2007.07.008 17767925

[22] Nakano R Edamura K Sugiya H Narita T Okabayashi K Moritomo T Evaluation of mRNA expression levels and electrophysiological function of neuron-like cells derived from canine bone marrow stromal cells. Am J Vet Res (2013) 74:1311–20. 10.2460/ajvr.74.10.1311 24066915

[23] Nakano R Edamura K Nakayama T Teshima K Asano K Narita T Differentiation of canine bone marrow stromal cells into voltage- and glutamate-responsive neuron-like cells by basic fibroblast growth factor. J Vet Med Sci (2015) 77:27–35. 10.1292/jvms.14-0284 25284120 PMC4349535

[24] Nakano R Edamura K Nakayama T Narita T Okabayashi K Sugiya H. Fibroblast growth factor receptor-2 contributes to the basic fibroblast growth factor-induced neuronal differentiation in canine bone marrow stromal cells via phosphoinositide 3-kinase/Akt signaling pathway. PloS One (2015) 10:e0141581. 10.1371/journal.pone.0141581 26523832 PMC4629880

[25] Konno T Nakano R Mamiya R Tsuchiya H Kitanaka T Namba S Expression and function of interleukin-1β-induced neutrophil gelatinase-associated lipocalin in renal tubular cells. PloS One (2016) 11:e0166707. 10.1371/journal.pone.0166707 27851800 PMC5112913

[26] Kitanaka N Nakano R Kitanaka T Kitanaka N Namba S Konno T NF-κB p65 and p105 implicate in interleukin 1β-mediated COX-2 expression in melanoma cells. PloS One (2018) 13:e0208955. 10.1371/journal.pone.0208955 30562372 PMC6298655

[27] Bradford MM A rapid and sensitive method for the quantitation of microgram quantities of protein utilizing the principle of protein-dye binding. Anal Biochem (1976) 72:248–54. 10.1016/0003-2697(76)90527-3 942051

[28] Smith WL DeWit DL Garavito RM Cyclooxygeneses: structural, cellular, and molecular biology. Annu Rev Biochem (2000) 69:145–82. 10.1146/annurev.biochem.69.1.145 10966456

[29] Simmons DL Botting RM Hla T Cyclooxygenese isozymes: the biology of prostaglandin synthesis and inhibition. Pharmacol Rev (2004) 56:387–437. 10.1124/pr.56.3.3 15317910

[30] Vermeulen L De Wilde G Notebaert S Vanden Berghe W Haegeman G Regulation of the transcriptional activity of the NF-κB p65 subunit. Biochem Pharmacol (2002) 64:963–70. 10.1016/S0006-2952(02)01161-9 12213593

[31] Viatour P Merville MP Bours V Chariot A Phosphorylation of NF-κB and IκB proteins: implications in cancer and inflammation. Trends Biochem Sci (2005) 30:43–52. 10.1016/j.tibs.2004.11.009 15653325

[32] Perkins ND Post-translational modifications regulating the activity and function of the NF-κB pathway. Oncogene (2006) 25:6717–30. 10.1038/sj.onc.1209937 17072324

[33] Huang B Yang XD Lamb A Chen LF Posttranslational modifications of NF-κB: another layer of regulation for NF-κB signaling pathway. Cell Signal (2010) 22:1282–90. 10.1016/j.cellsig.2010.03.017 PMC2893268 20363318

[34] Christian F Smith EL Carmody RJ The regulation of NF-κB subunits by phosphorylation. Cells (2016) 5:E12. 10.3390/cells5010012 26999213 PMC4810097

[35] Cartwright T Perkins ND Wilson CL NFKB1: a suppressor of inflammation, ageing and cancer. FEBS J (2016) 283:1812–22. 10.1111/febs.13627 26663363

[36] Kyriakis JM Avruch J Mammalian mitogen-activated protein kinase signal transduction pathways activated by stress and inflammation. Physiol Rev (2001) 81:807–69. 10.1152/physrev.2001.81.2.807 11274345

[37] Kyriakis JM Avruch J Mammalian MAPK signal transduction pathways activated by stress and inflammation: a 10-year update. Physiol Rev (2012) 92:689–737. 10.1152/physrev.00028.2011 22535895

[38] Akaogi J Nozaki T Satoh M Yamada H Role of PGE2 and EP receptors in the pathogenesis of rheumatoid arthritis and as a novel therapeutic strategy. Endocr Metab Immune Disord Drug Targets (2006) 6:383–94. 10.2174/187153006779025711 17214584

[39] Park JY Pillinger MH Abramson SB Prostaglandin E_2_ synthesis and secretion: the role of PGE_2_ synthases. Clin Immunol (2006) 119:229–40. 10.1016/j.clim.2006.01.016 16540375

[40] Bonizzi G Karin M The two NF-κB activation pathways and their role in innate and adaptive immunity. Trends Immunol (2004) 25:280–8. 10.1016/j.it.2004.03.008 15145317

[41] Chen FE Kempiak S Huang DB Phelps C Ghosh G Construction, expression, purification and functional analysis of recombinant NFκB p50/p65 heterodimer. Protein Eng (1999) 12:423–8. 10.1093/protein/12.5.423 10360983

[42] Phelps CB sengchanthalangsy LL Huxford T Ghosh G Mechanism of IκBα binding to NF-κB dimers. J Biol Chem (2000) 275:29840–6. 10.1074/jbc.M004899200 10882738

[43] Tak PP Firestein GS NF-κB: a key role in inflammatory diseases. J Clin Invest (2001) 107:7–11. 10.1172/JCI11830 11134171 PMC198552

[44] Hoesel B Schmid JA The complexity of NF-κB signaling in inflammation and cancer. Mol Cancer (2013) 12:86. 10.1186/1476-4598-12-86 23915189 PMC3750319

[45] Tieri P Termanini A Bellavista E Salvioli S Capri M Franceschi C Charting the NF-κB pathway interactome map. PloS One (2012) 7:e32678. 10.1371/journal.pone.0032678 22403694 PMC3293857

