# Supplementary material for: Non-Transcriptional and Translational Function of Canonical NF-κB Signaling in Activating ERK1/2 in IL-1β-Induced COX-2 Expression in Synovial Fibroblasts
Source: Front Immunol. 2020 Oct 7;11:579266. doi: 10.3389/fimmu.2020.579266 (PMC7576893; doi:10.3389/fimmu.2020.579266)
Supplement: Supplementary file 1 [file DataSheet_1.pdf]

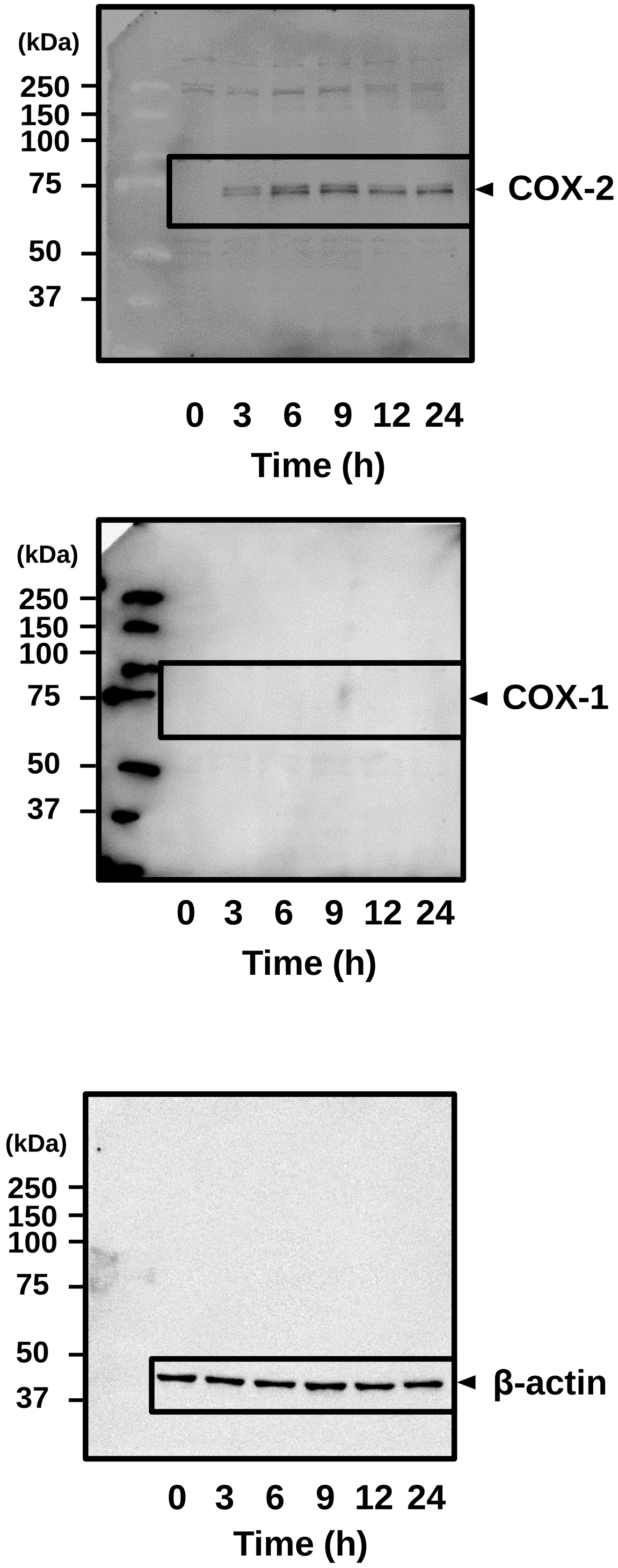

Fig. S1. Uncropped images for the blots shown in Fig. 1.

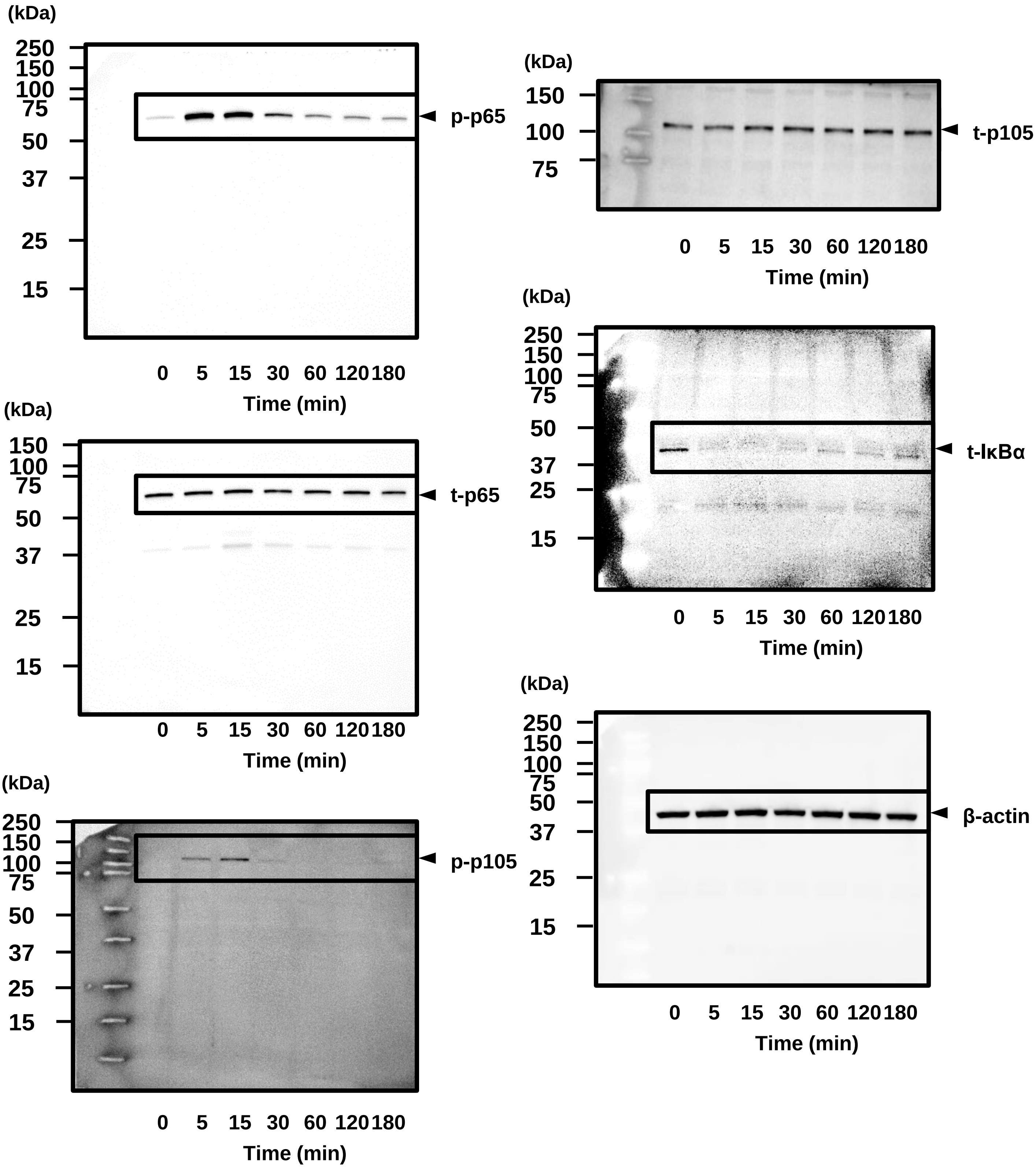

Fig. S2. Uncropped images for the blots shown in Fig. 3.

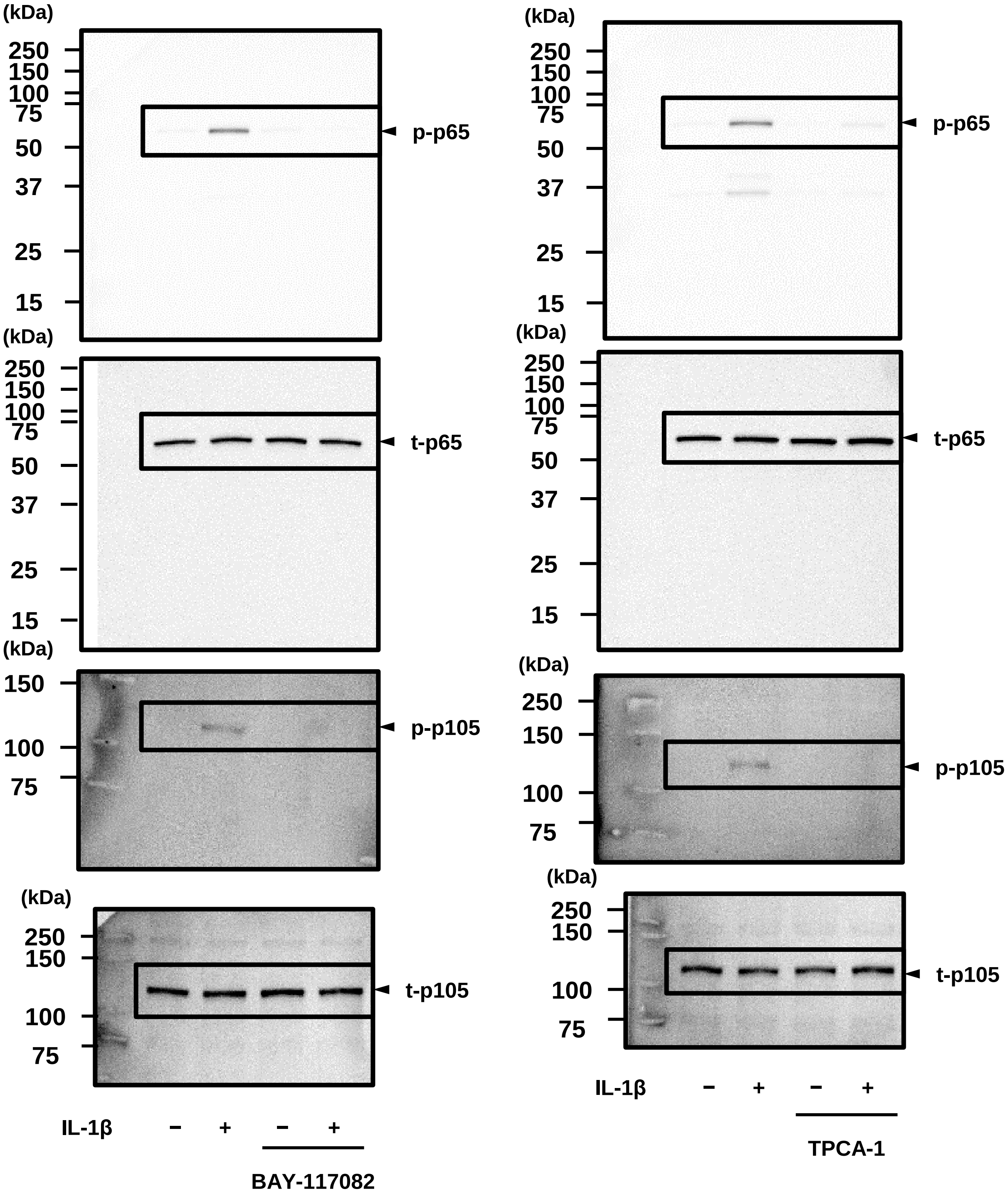

Fig. S2. Uncropped images for the blots shown in Fig. 3. (continue)

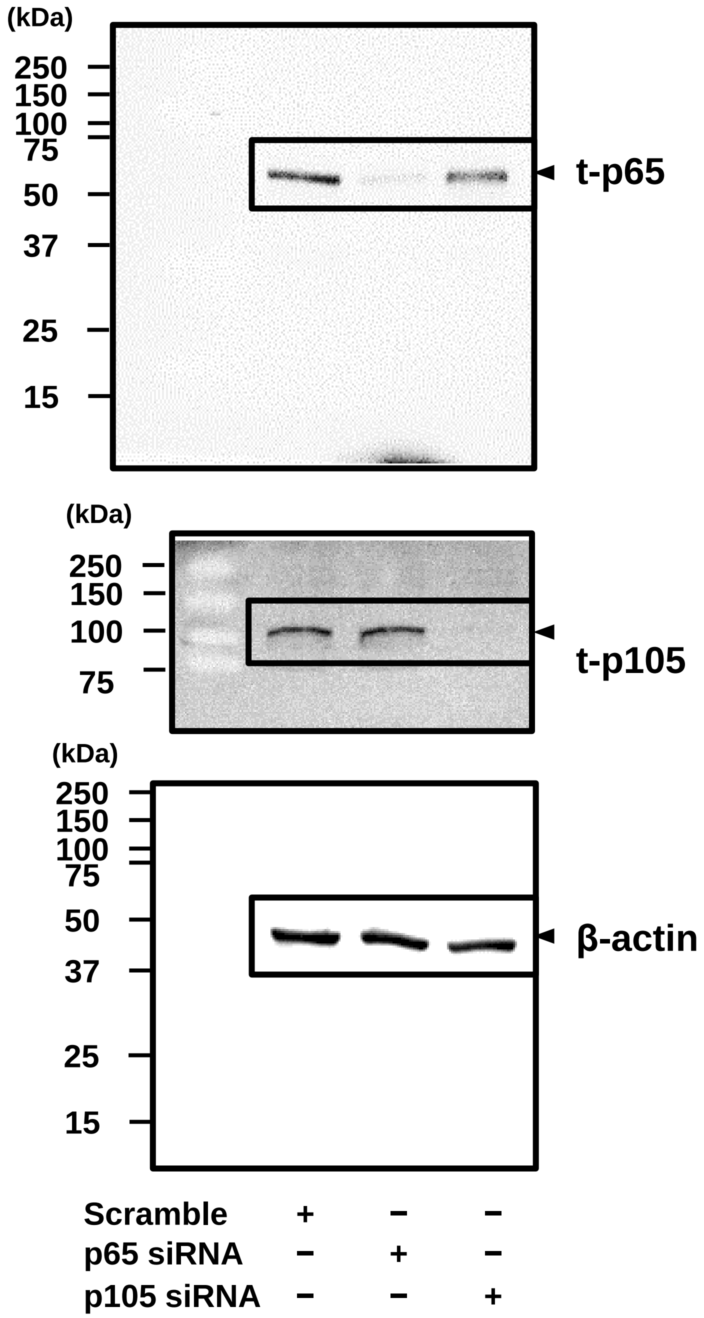

Fig. S3. Uncropped images for the blots shown in Fig. 4.

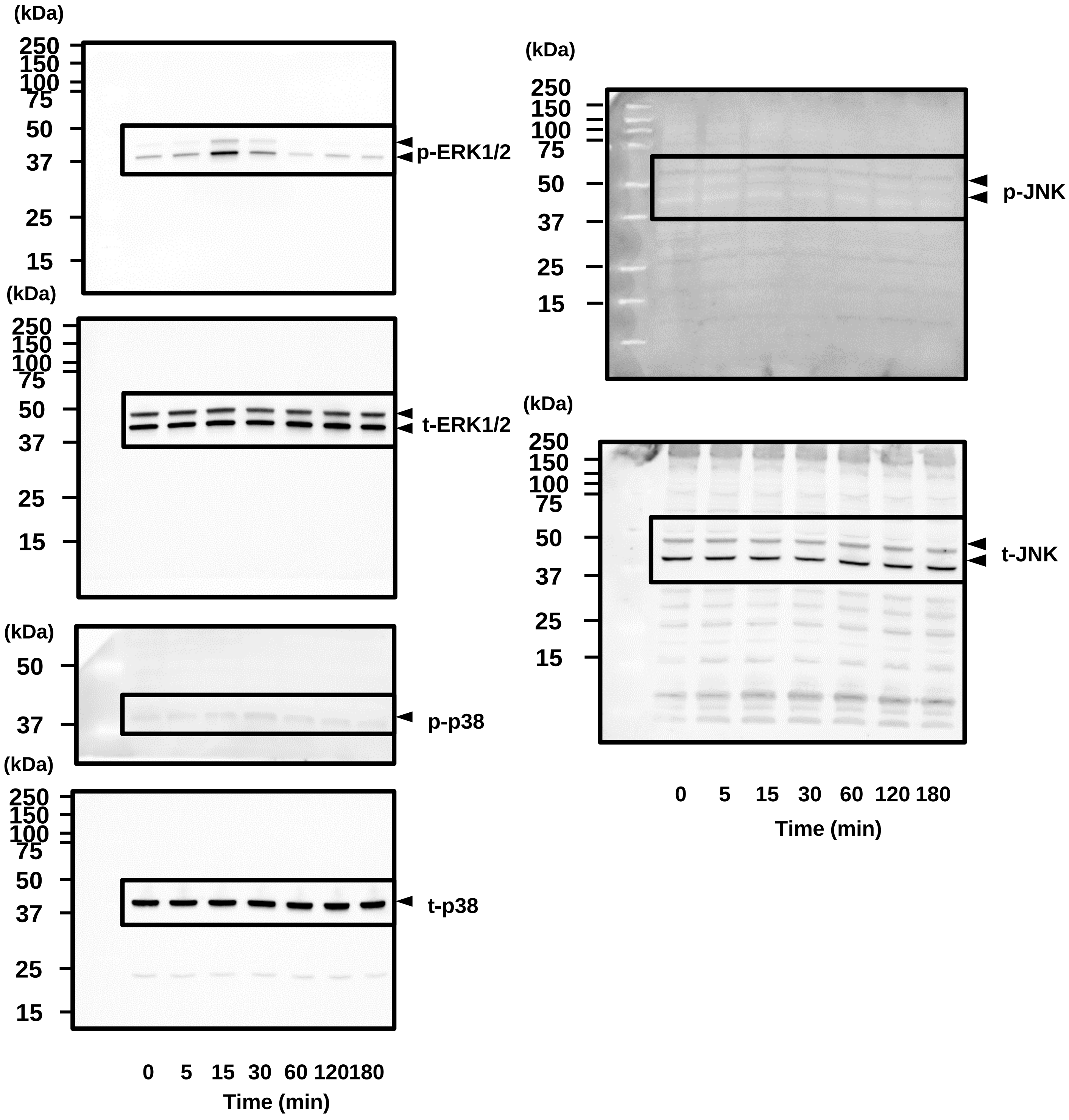

Fig. S4. Uncropped images for the blots shown in Fig. 6.

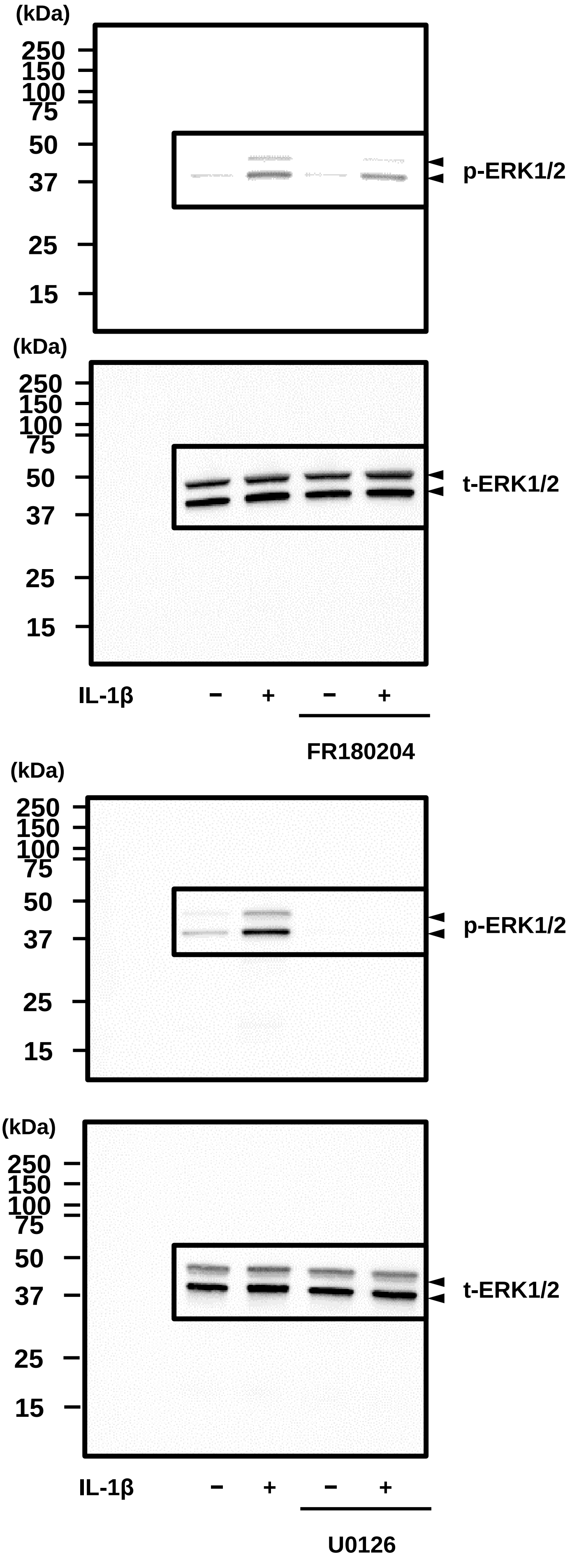

Fig. S4. Uncropped images for the blots shown in Fig. 6. (continue)

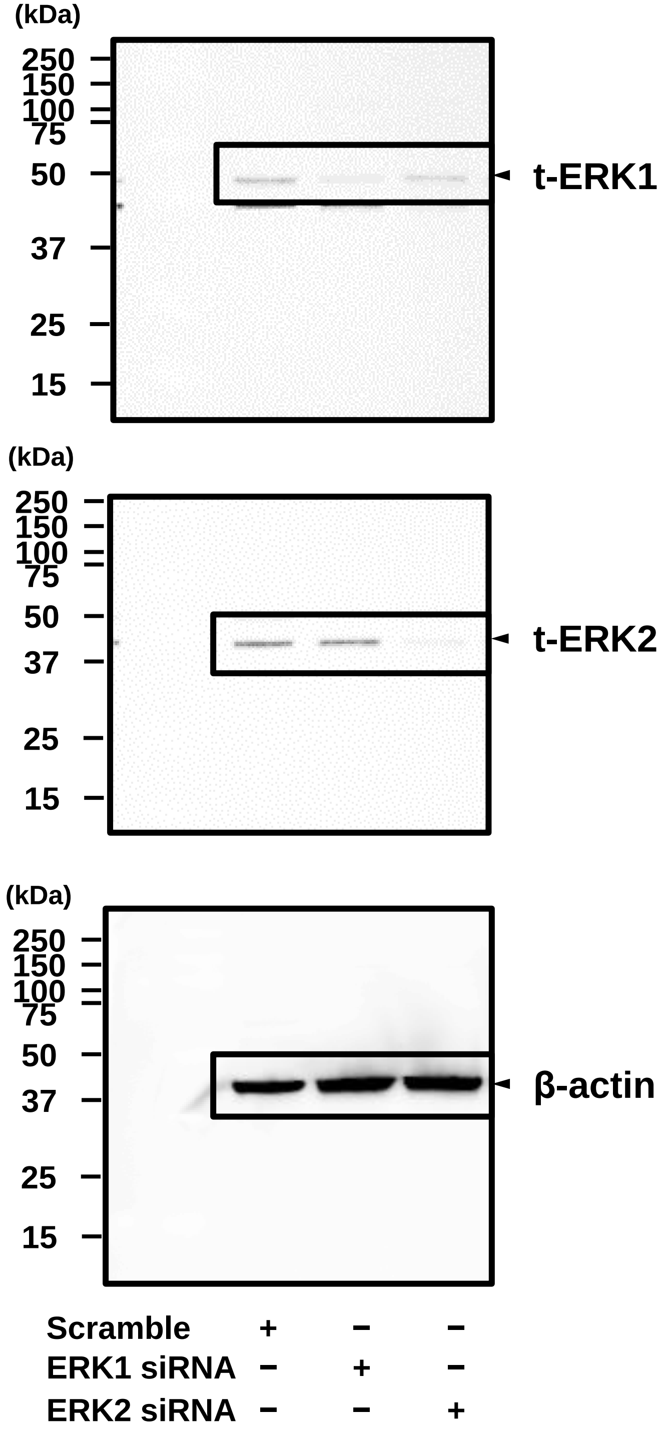

Fig. S5 Uncropped images for the blots shown in Fig. 7.

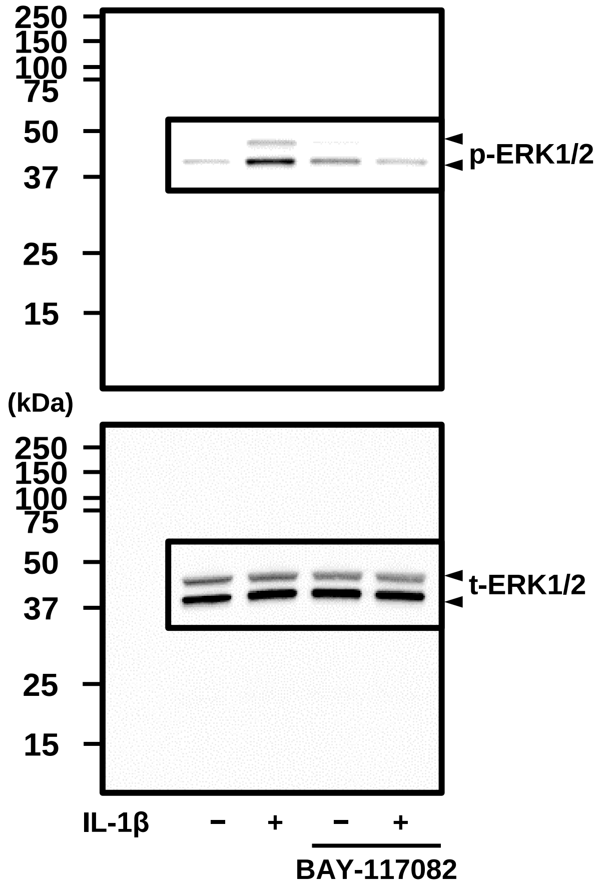

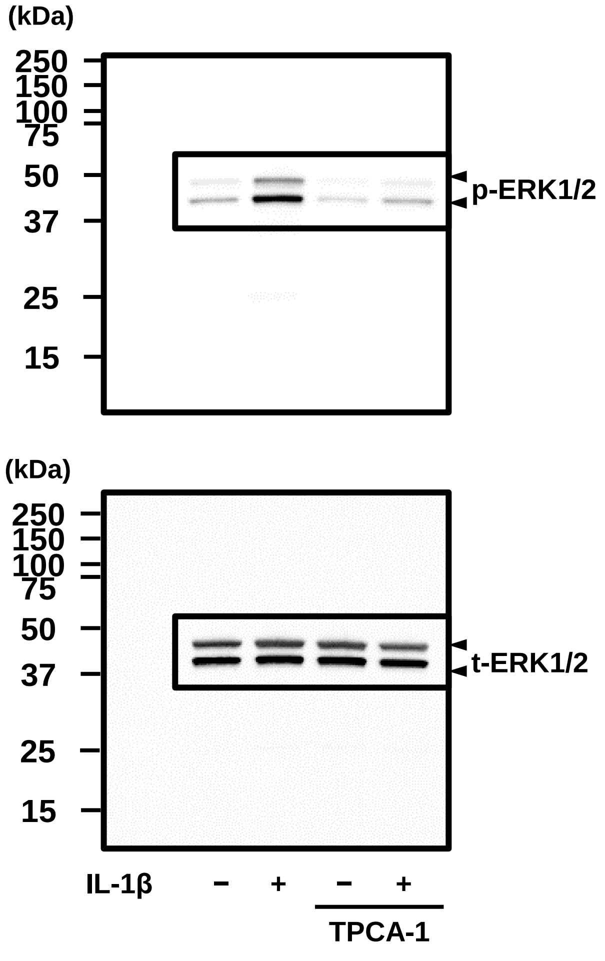

Fig. S6 Uncropped images for the blots shown in Fig. 8.

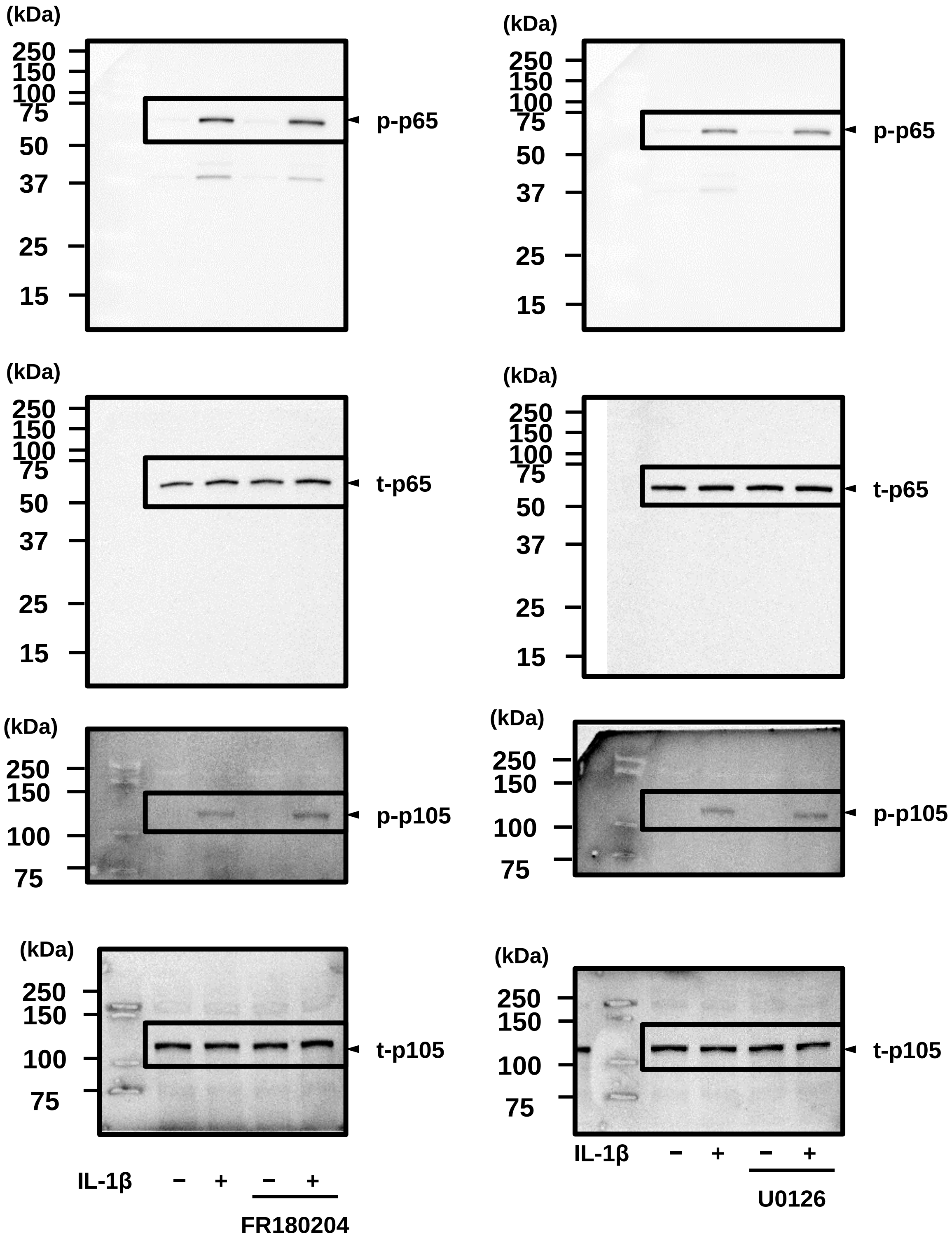

Fig. S6 Uncropped images for the blots shown in Fig. 8. (continue)

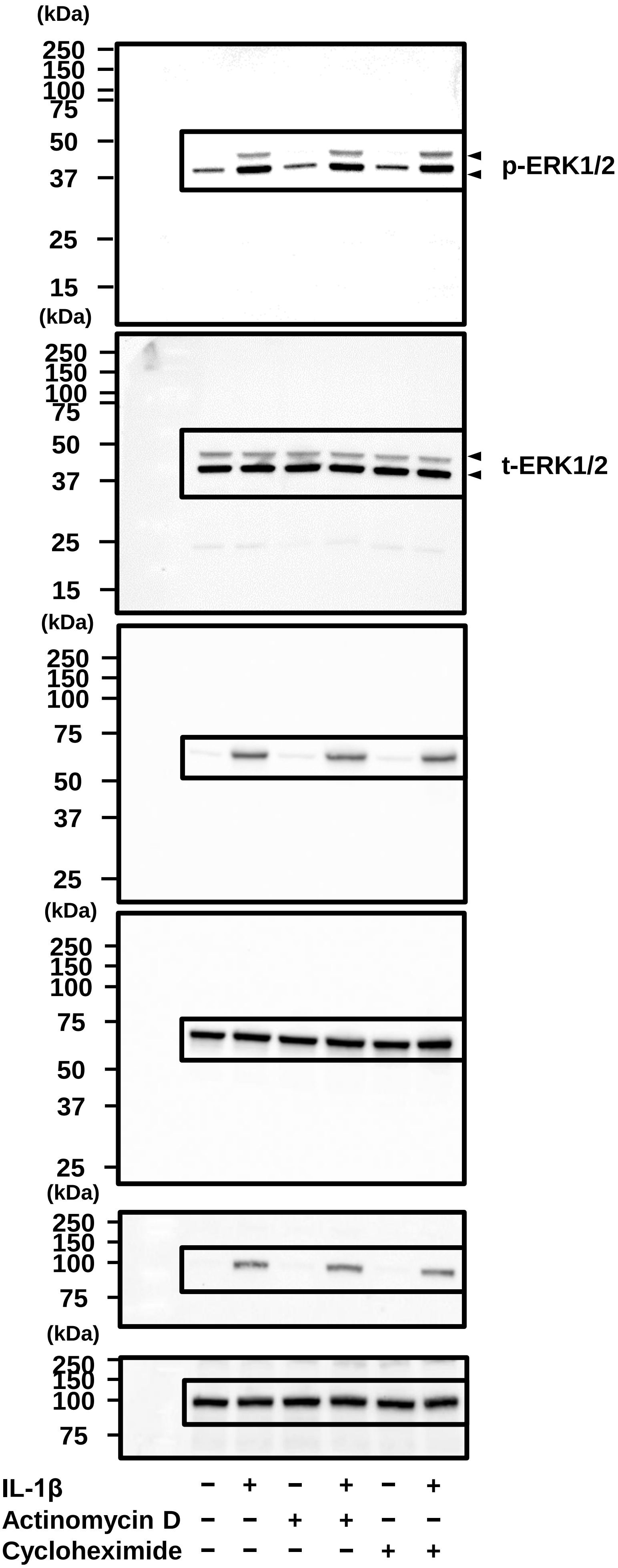

Fig. S7 Uncropped images for the blots shown in Fig. 9.

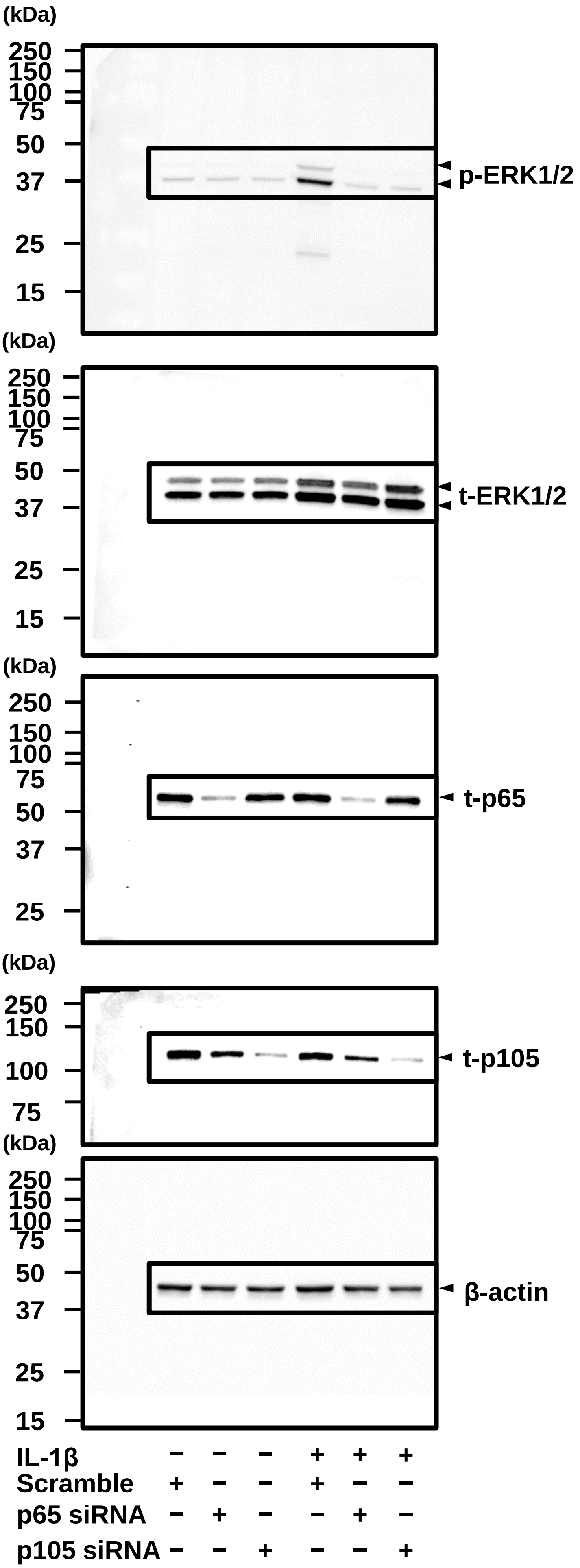

Fig. S7 Uncropped images for the blots shown in Fig. 9. (continue)

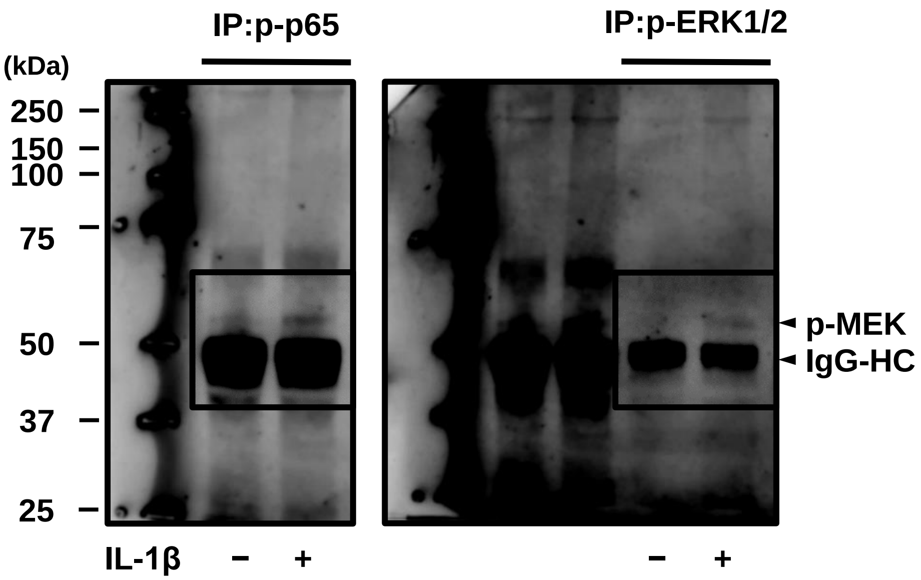

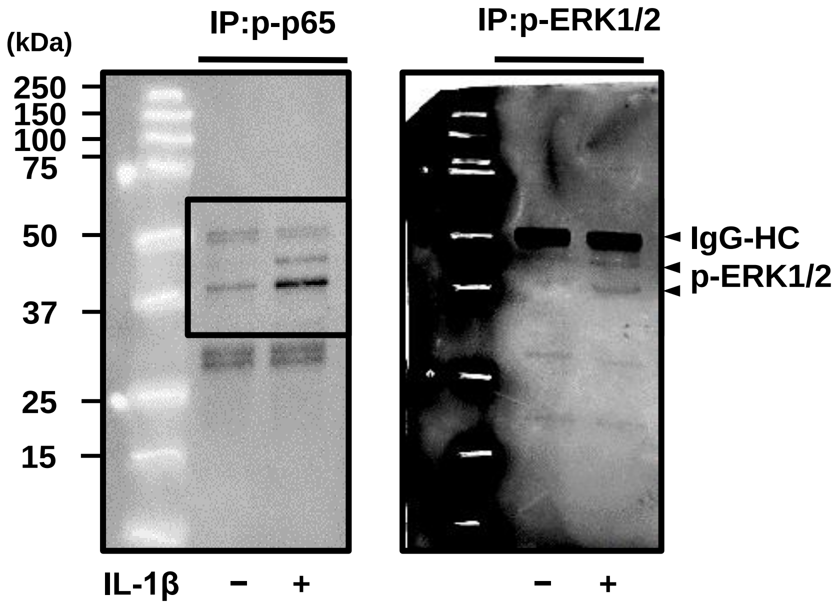

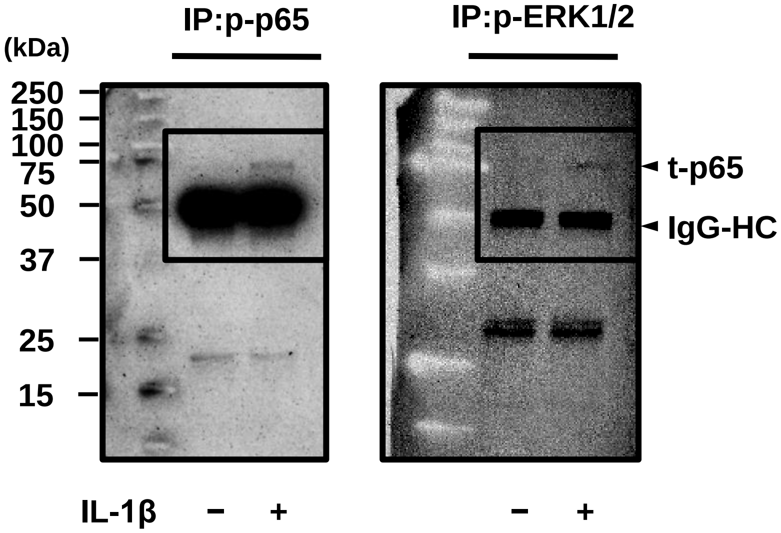

Fig. S7 Uncropped images for the blots shown in Fig. 9. (continue)
